# Predictors of new persistent opioid use after surgery in adults

**DOI:** 10.1007/s44254-024-00083-1

**Published:** 2025-01-17

**Authors:** Kathryn H. Gessner, John S. Preisser, Emily Pfaff, Rujin Wang, Kellie Walters, Robert Bradford, Marshall Clark, Mark Ehlers, Matthew Nielsen

**Affiliations:** 1 Department of Urology, University of North Carolina at Chapel Hill, Chapel Hill, NC 27599 USA; 2 Department of Biostatistics, Gillings School of Global Public Health, University of North Carolina at Chapel Hill, Chapel Hill, NC 27599 USA; 3 North Carolina Translational and Clinical Sciences Institute, University of North Carolina at Chapel Hill, Chapel Hill, NC 27599 USA; 4 Department of Medicine, University of North Carolina at Chapel Hill, Chapel Hill, NC 27599 USA

**Keywords:** Post-operative pain, Opioid dependency, Prediction modeling, Monte-Carlo cross-validation, Semantic technology

## Abstract

**Purpose:**

Persistent opioid use is one of the most common post-operative complications. Identification of at-risk patients pre-operatively is key to reducing post-operative opioid use. We sought to develop a predictive model for persistent post-operative opioid used and to determine if geographic factors from community databases improve model prediction based solely on electronic health records (EHRs) and claims data.

**Methods:**

EHR and claims data for 4,116 opioid-naïve surgical patients older than 18 in North Carolina were linked with census tract-level unemployment data from the American Community Survey and Centers for Disease Control and Prevention data on opioid prescriptions and deaths attributed to drug poisoning. Primary outcome was new persistent opioid use and covariates included patient factors from EHR, claims data, and geographic factors. Multivariable logistic regression models of potential risk factors were evaluated.

**Results:**

6.0% of patients developed new persistent opioid use. Associated risk factors based on multivariable logistic regressions include age (adjusted odds ratio [AOR] 1.08; 95% confidence interval [CI] 1.00, 1.16), back and neck pain (1.82; 1.39, 2.39), joint disorders (1.58; 1.18, 2.11), mood disorders (1.71; 1.28, 2.28), opioid retail prescription (1.04; 1.00, 1.07) and drug poisoning rates (1.33; 1.09, 1.62). On Monte-Carlo cross-validation, the addition of geographic factors to EHRs and claims may modestly improve prediction performance (area under the curve, AUC) of logistic regression models compared to those based on EHRs and claims data (AUC 0.667 (95% CI 0.619, 0.717) vs AUC 0.653 (0.600, 0.706)).

**Conclusions:**

Co-morbidities and area-based factors are predictive of new persistent post-operative opioid use. As the addition of geographic-based factors did not significantly improve performance of multivariable logistic regression, larger samples are needed to fully differentiate models.

**Supplementary Information:**

The online version contains supplementary material available at 10.1007/s44254-024-00083-1.

## Introduction

New persistent opioid use following surgery has been identified as one of the most common postoperative complications, affecting 6–10% of opioid-naïve surgical patients [1–4]. Pre-operative identification of patients who are at high risk of developing persistent use could allow for interventions to reduce post-operative opioid use, including improved pre-operative counseling, use of opioid-sparing pathways, and the use of mobile applications guiding patients in post-operative pain management [5, 6]. Such identification relies on screening patients for risk factors associated with persistent opioid use, which are not well-defined. Consistent across multiple studies, prior opioid, benzodiazepine, and antidepressant use are associated with a higher level of persistent opioid use following surgery [7–10]. Specifically, pre-operative benzodiazepine use is associated with increased long-term opioid use and higher post-operative opioid dosages [10], possibly reflecting the association between persistent post-operative pain with depression and stress [11]. Additionally, the specific type of surgery may impact a patient’s risk of developing post-operative use, although different studies demonstrate conflicting associations [2, 7, 9]. Additionally, studies are conflicting in whether male patients vs female patients or older vs younger patients are at higher risk [7–9, 12] and no standardized risk stratification tool exists to predict a patient’s risk based on multiple factors.

Therefore, given the lack of definitive risk factors and risk stratification tools, it is difficult to determine pre-operatively which patients will develop persistent opioid use following surgery. Additionally, while individual patient factors, such as age or medical comorbidities, may predict their risk of persistent post-operative opioid use, we hypothesize that ecological social or geographic factors, including local opioid prescription rates or deaths due to opioid toxicity, may also impact a patient’s risk. Specifically, higher opioid prescriptions are associated with county-level factors, including higher unemployment [13], and higher levels of prescription opioid overdoses are present in more economically disadvantaged zip codes [14]. There is a clear need for improved pre-operative risk stratification with incorporation of both patient and contextual factors to identify high risk patients. Previous studies focusing on opioid use risk factors have utilized electronic health records (EHRs), claims, prescription, and vital statistics data [7–9, 12], but these data do not capture factors unrelated to an individual’s health conditions.

Although the EHR is a robust data resource, we hypothesized that the complexities surrounding opioid use would be better captured by linking of disparate data sets for health behaviors and risk factors not found in the EHR [1, 8]. Efforts to inform evidence-based opioid prescribing practices exemplify the value of linking EHR data to non-clinical data: EHR data can be combined with other datasets such as insurance claims, environmental data, neighborhood-level data, or socioeconomic status data to generate a more holistic picture of a patient’s risk of developing new persistent opioid use. In countries with national healthcare systems and national databases, such as New Zealand, this data linkage has been successfully performed in a large population-based study of persistent opioid use after surgery [15]. However, such data linkages are a methodological challenge [16–18], particularly when the datasets are large, highly varied in structure, and aren’t linked on an individual patient level. To address the methodological challenge of linking large and disparate datasets, we developed a Resource Description Framework (RDF) triplestore as a hub for combining relevant data [19]. A triplestore RDF is a specialized database that stores data as triples, each consisting of a subject, predicate, and object [19]. The rigidity of this data structure allows for data interconnectivity and complex queries based on logical reasoning [19]. Triplestores themselves are not new and have been implemented in the bioinformatics field [20–23], but are rarely used with EHR data. However, given the dense relationships present within clinical data and the many possible sources of patient data, we hypothesized that a triplestore RDF would optimally link disparate databases to allow for complex queries of clinical data [19].

The primary objective of this retrospective study utilizing EHRs, claims data and linked community databases to synthesize the RDF triplestore was to develop and validate a predictive model for opioid dependency following surgery and to determine if ecological variables improve prediction of persistent opioid use following surgery compared to a model solely based on EHRs and claims. A secondary objective was to describe the relative contributions of claims and EHR data for the identification of patient co-morbid conditions, which are among the candidate predictors of new persistent opioid use.

## Materials and methods

### Patient cohort and EHR data

The patient cohort consists of 4,159, opioid-naïve surgical patients age 18 and older with index surgery date between 04/14/2014 to 03/31/2017, insured by a large private commercial insurer in the Southeast, and meeting inclusion criteria. Specifically, we defined a cohort of opioid naïve surgical patients as patients who filled ≥ 1 opioid prescriptions either in the month before or within two weeks after discharge, but *did not* fill an opioid prescription 12 months to 31 days prior to surgery. We excluded patients with an opioid use disorder or who underwent additional surgery in the six-month postoperative period. The source for EHR clinical data was the Carolina Data Warehouse for Health (CDWH), the UNC Health’s enterprise clinical data warehouse. From the CDWH, we recorded patient gender, age at surgery, race (black, white, other), ethnicity (Hispanic/Not Hispanic), and surgery type (Current Procedural Terminology code). Smoking status, benzodiazepine use, and pain score data were defined and extracted from the CDWH as described in the Supplemental Methods. Missing data was imputed as described as below and in the Supplemental Methods. Briefly, single imputation was used to impute missing data for race and “ever smoker”, with conditional mode imputation used for race and unconditional mode imputation used for “ever smoker”. For missing pain scores, multiple imputation using ordinal logistic regression imputation based on cumulative logits and assuming proportional odds was fitted for pain as a function of patient-level factors.

### Mental health and physical pain EHR and claims data

A deterministic match at the patient level was used to extract information on mental health, physical pain and benzodiazepine use from the EHR master file and separate claims data sources. Subcategories of mental health diagnoses were created by combining disorder types as previously described [2]: mood disorders (adjustment disorders, anxiety disorders and mood disorders), suicidality (suicide and self-harm), disruptive behavior disorders (attention deficit, conduct and disruptive disorders, impulse control disorders), personality disorders (personality disorders and schizophrenia), substance use disorders (substance use disorders and alcohol related disorders), and miscellaneous disorders. Specifically, using International Classification of Diseases (ICD)-9 and ICD-10 codes (Supplemental Table 1) corresponding to an a priori defined set of specified mental health conditions, we identified encounters within a visit window from one year prior to surgery through the surgical index date. We deleted duplicate records within patients and merged datasets to remove duplications across ICD-9 and ICD-10 codes, which resulted in 1,613 patients with one or more designated mental health condition. Using similar data manipulation methodology and an a priori defined set of ICD-9 and ICD-10 codes corresponding to “back and neck pain” and “joint disorder” diagnoses, we identified 2,646 patients with these disorders. Finally, the described mental health and physical pain datasets were linked to form a single dataset containing the dichotomous condition variables indicating the presence or absence of each condition within the year prior to surgery (Supplemental Fig. 1).

### Community dataset of geographic area factors

Census tract unemployment rates (percentage, 0 to 100) from the 2016 American Community Survey (ACS) were assigned to patients based on their address. County-level data were obtained from the Centers for Disease Control and Prevention (CDC), which included 1) rates of the number of opioid retail prescriptions per 100 persons in 2015 [24] and 2) deaths attributed to drug poisoning per 100,000 population in 2016 [25]. While linking CDC opioid retail prescription rate using patient address may not reflect prescribing patterns of each patient’s individual surgeon, the goal of this study was to evaluate geographic, community-oriented factors which impact a patient’s risk factor for persistent opioid use. Evaluating county-level prescribing patterns instead of clustering based on the patient’s specific provider allows assessment of the impact of geographic community factors on persistent opioid use. The county-level drug poisoning rates are available as 13 categories (Supplemental Table 2); to preserve the ordinal nature of the data, we used interval midpoints for modeling, with 31.05 assigned to the last category of “ > 30”.

### Data linkage and outcome variable

**Fig. 1:**
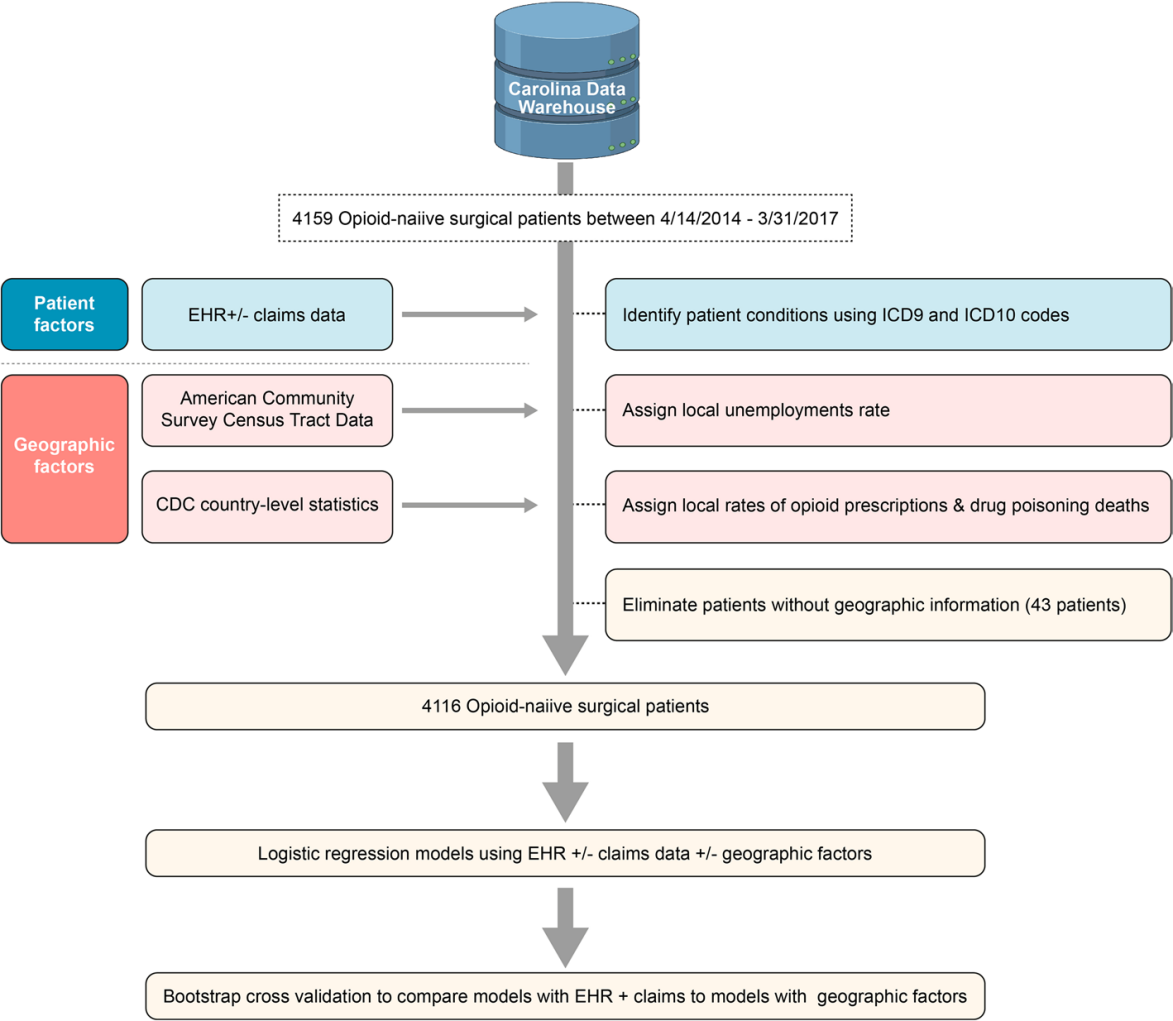
Experimental design outlining data linkage procedure and creation of Triplestore dataset

The dataset of mental health, pain conditions, and benzodiazepine use was merged with the master EHR data set of 4,159 patients and the geographic community dataset into an integrated Triplestore RDF as previously described [19] (Fig. 1). Patients that could not be linked with the community dataset due to having unknown county or census tract identifier were excluded from the analysis (43 patients). Our primary outcome was new persistent opioid use, as defined in previous studies as at least one opioid prescription filled between 90 and 180 days after surgery among previously opioid-naïve patients [2, 9]. A secondary objective of the study was to ascertain the relative contributions of claims and EHR data for the identification of patient co-morbid conditions and benzodiazepine use and prediction of new opioid use.

### Ethics statement

This study with a waiver of informed consent was approved by the University of North Carolina at Chapel Hill institutional review board (approval no. 18–1154). To optimize reporting of this study, we used the TRIPOD checklist when writing our report [26].

### Statistical analysis

Demographic characteristics (gender, race, ethnicity, age), pain scores, and mental and physical pain conditions were summarized for the entire sample. Pearson Chi-square tests were performed to test associations between persistent opioid use and demographic/condition variables. For uncommon conditions (suicidality, personality disorders), Fisher’s exact test was used. The Wilcoxon Rank Sum Test was used to compare pain scores between persistent opioid users and non-persistent opioid users. Frequencies for co-morbid conditions and benzodiazepine use were calculated based on EHR alone to assess the relative contribution of EHR vs claims and EHR in identifying these variables.

We evaluated multivariable logistic regression models of potential risk factors to predict the risk of new persistent opioid use. Medical conditions less than 10% prevalence were not included in the models due to the small number of patients with these conditions and who were new opioid users. An initial multivariable logistic regression predictive model (E + C) of new persistent opioid use based solely on EHR and claims data was fitted with the following variables: gender, race, age, ever being a smoker, mean pain score, benzodiazepine use, and dichotomous indicators for comorbid medical conditions meeting the prevalence threshold. A second model (E + C + G) additionally included the three geographic-area factors (unemployment rate, rate of opioid retail prescriptions and drug poison death rate). For the secondary objective, a third model based solely on claims data having the same predictors as Model E + C was also fitted.

With regards to variable selection, statistical considerations based on sample size limited the number of variables in the models. A common rule-of-thumb is that there should be at least 10 observations for the rarer outcome (new opioid user, in this case) per parameter being considered in the model [27]. Therefore, as there are 245 new opioid users in the dataset, application of this rule allows up to 24 parameters in the model and this was considered during variable selection.

### Assessment of prediction performance

For each model, sensitivity, specificity, false-positive rate and false negative rates were calculated using the observed proportion of incident persistent opioid use as the empirically determined threshold. To evaluate prediction performance, subject-level model-predicted probabilities of persistent opioid use were compared to the threshold. Additionally, for all possible cutoff points, we calculated the area under the curve (AUC) of receiver operating characteristic (ROC) curve (AUC) for each model. To better assess model performance, estimates of monte-carlo cross-validated prediction accuracy were obtained [28]. Specifically, a random sample without replacement of two-thirds of the overall sample of opioid-naïve patients (*n* = 2,744) was fitted with a logistic regression model, obtaining the estimated regression coefficients from the Monte-Carlo replicate training dataset. Using the fitted logistic regression, the predicted values (*n* = 1,372) of the response for each of the patients not selected into the training set were determined and the fit statistics (AUC, Brier score) were calculated. The Brier score is the average of the squared distance between the patient’s observed status and the predicted probability such that a lower score is better. The averages of the fit statistics from the 500 samples were computed with their Monte-Carlo percentile confidence intervals. The cross-validated ROC curve was obtained by averaging across the Monte-Carlo sample estimates of sensitivity for each value of 1 minus specificity. For each model, a single imputation step for pain score was added during each Monte-Carlo replicate [29] using multinomial logistic regression as described Supplemental Methods.

## Results

**Fig. 2:**
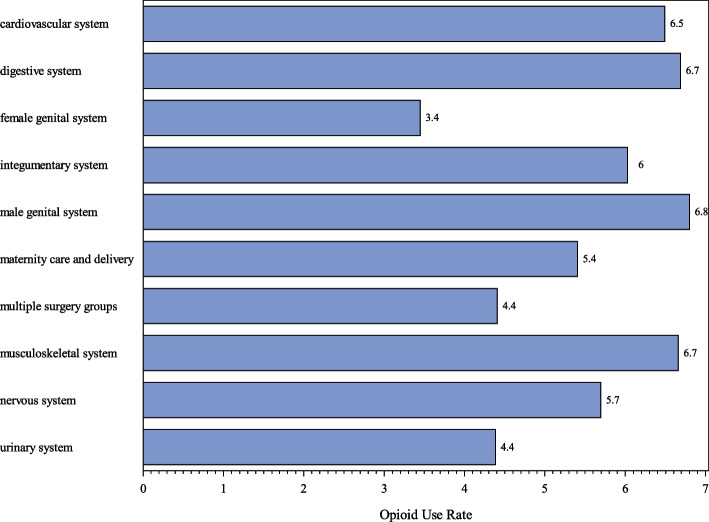
Incidence of new persistent opioid use by surgery procedure groups (＞100 per group)

The 4,116 patients in the final dataset had mean age of 44.6 years; 55.0% were female and 45.0% were male; 81.2% were white and 11.1% were black; and 89.8% were non-Hispanic whereas 4.2% were Hispanic (with 6.0% missing) (Table 1). The 219 patients (5.3%) with missing race were all assigned as white, the modal category in all 48 groups described in the Supplemental Material for the imputation. Six patients with unknown smoking status were imputed as “never smoke”. Pain scores were missing for 946/4,116 (23.0%) of study participants. However, model outcomes and predictor variables were unchanged when this variable was included in models with imputation or excluded. Inclusion allowed for multivariable adjusted association between pain scores and persistent opioid use, which was positive but not statistically significant. Overall, 6.0% of patients developed new persistent opioid use with similar rates across surgery subgroups (Fig. 2, Supplemental Table 3).

**Table 1:** Patient characteristics^a^ and incidence of persistent opioid use 90 to 180 post-surgery (*n* = 4,116)

	Total number (column percent)	Persistent opioid use N, (% for row subgroup)^b^	*p*-value^c^
Gender			0.33
Female	2,262 (55.0%)	142 (6.3%)	
Male	1,854 (45.0%)	103 (5.6%)	
Race			0.47
Black	455 (11.1%)	31 (6.8%)	
White	3,342 (81.2%)	199 (6.0%)	
Other	319 (7.8%)	15 (4.7%)	
Ever smoker			0.0025
No	2,749 (66.8%)	142 (5.2%)	
Yes	1,367 (33.2%)	103 (7.5%)	
Benzodiazepine Use			0.0012
No	3,640 (88.4%)	201 (5.5%)	
Yes	476 (11.6%)	44 (9.2%)	
Back and neck pain			< .0001
No	2,940 (71.4%)	131 (4.5%)	
Yes	1,176 (28.6%)	114 (9.7%)	
Joint disorders			< .0001
No	1,865 (45.3%)	74 (4.0%)	
Yes	2,251 (54.7%)	171 (7.6%)	
Mood disorders			< .0001
No	2,892 (70.3%)	138 (4.8%)	
Yes	1,224 (29.7%)	107 (8.7%)	
Suicidality			0.076^*^
No	4,099 (99.6%)	242 (5.9%)	
Yes	17 (0.4%)	3 (17.7%)	
Disruptive behavior disorders			0.72
No	3,885 (94.4%)	230 (5.9%)	
Yes	231 (5.6%)	15 (6.5%)	
Personality disorders and schizophrenia			0.26^*^
No	4,086 (99.3%)	245 (6.0%)	
Yes	30 (0.7%)	0 (0%)	
Substance use disorders			0.61
No	3,798 (92.3%)	224 (5.9%)	
Yes	318 (7.7%)	21 (6.6%)	
Miscellaneous disorders			0.24
No	3,912 (95.0%)	229 (5.9%)	
Yes	204 (5.0%)	16 (7.8%)	

^a^Based on EHRs and claims. ^b^The percentages are the percent opioid use for the subgroup in each row. ^c^Tests of whether patient characteristic or condition type is associated with new persistent opioid use. ^*^Fisher’s exact test, otherwise Pearson chi-square test

Based on bivariate tests between patients with and without persistent opioid use, patients who were ever smokers, used benzodiazepines, or had back and neck pain, joint disorder, and mood disorders were more likely to develop persistent opioid user than patients without those characteristics (Table 1). Mean pain score was 3.8 among those who developed persistent opioid use versus 3.2 among patients who did not (*p* < 0.0001). There were no statistically significant associations between persistent opioid use and race; ethnicity (not shown); or having a diagnosis of disruptive behavior disorder, personality disorder, substance use disorder, and miscellaneous disorder prior to surgery (Table 1).

Descriptive data suggest a possible trend for increasing percentage of new persistent opioid use with higher community levels of unemployment rate, opioid prescription rate and drug poisoning rates (Table 2). Exceptions to these trends were for the highest categories of prescription and drug poison rates corresponding to upper quartiles of counties where sample sizes of patients were lowest. Note that the distribution of study participants underrepresents individuals from geographic areas that are most impacted by unemployment, high opioid retail prescription rates and high drug poison death rates. For example, only 12.4% of study participants are from the 43 counties with CDC opioid retail rates exceeding 89.6 prescriptions per 100 persons (in 2015) whereas 33% of all residents in the eighty-eight county region live in these higher opioid retail rate counties (Table 2).

**Table 2:** Population and sample distribution of area^a^ characteristics for the sample of opioid-naïve surgical patients (*n* = 4,116) and number (%) of study participants who develop persistent opioid use by area characteristic

Area characteristic		Size of population in areas with characteristic (column %)^b^	Size of study samples in areas with the characteristic ( column %)^c^	Number (%) of study participants with persistent opioid use in areas with the characteristic^d^
Unemployment Rate (percentage)	Number of Census Tracts (*n* = 946)			
< 5.0	284	1,093,172 (29.5%)	1,583 (38.5%)	74 (4.7%)
5.0 to 7.0	198	815,457 (22.0%)	1,066 (25.9%)	72 (6.8%)
7.0 to 10.0	219	864,787 (23.4%)	808 (19.6%)	52 (6.4%)
> 10.0	245	928,754 (25.1%)	659 (16.0%)	47 (7.1%)
CDC Opioid Retail Prescriptions Rate (per 100 persons)	Number of Counties (*n* = 88)			
< = 76	23	3,287,246 (44.0%)	2,979 (72.4%)	160 (5.4%)
76–89.6	22	1,723,792 (23.0%)	627 (15.2%)	48 (7.7%)
89.6–111.5	22	1,256,084 (16.8%)	282 (6.9%)	24 (8.5%)
> 111.5	21	1,211,526 (16.2%)	228 (5.5%)	13 (5.7%)
CDC Drug Poison Death Rate (per 100,000 persons)	Number of Counties (*n* = 88)			
6.1–12	17	2,201,699 (29.4%)	2,151 (52.3%)	108 (5.0%)
12.1–16	26	2,502,970 (33.5%)	1,384 (33.6%)	91 (6.6%)
16.1–22	25	1,737,068 (23.2%)	388 (9.4%)	35 (9.0%)
> 22	20	1,036,911 (13.9%)	193 (4.7%)	11 (5.7%)

^a^The sample of surgical patients resided in 946 census tracts and 88 counties. This column gives the number and percentage of areas (census tracts or counties) in each category of the area characteristic. ^b^The second columns gives numbers and percentages for the distribution of the *population* 18 years and older across groups (rows) of census tracts (data source: American Community Survey) or counties (data source: Centers for Disease Control and Prevention county-level statistics) as applicable. ^c^The third column gives the distribution of surgical patients in the *sample* across the groups defined by the area characteristics, for comparison with the second column. ^d^For each group (row), the denominator for the percent persistent opioid use in the sample of surgical patients is the corresponding group sample size n in the third column

Preliminary descriptive analysis identified three comorbidities having prevalence exceeding 10%: mental health/physical pain conditions of mood disorders, back & neck pain, and joint disorders; these were included in the multivariable logistic regression models for new persistent opioid use (Table 3). In Model E + C, risk factors predictive of new persistent opioid use were back and neck pain (adjusted odds ratio [AOR], 1.86; 95% confidence interval [CI] 1.42, 2.44), joint disorders (aOR 1.57; 95% CI 1.17, 2.10), and mood disorders (aOR 1.69; 95% CI 1.27, 2.26). In Model E + C + G, new persistent opioid use is statistically significantly associated with higher county-level opioid retail prescription rates and drug poisoning rates. Additionally, model E + C + G had slightly better, but non-significant, prediction performance (AUC 0.667; 95% CI 0.619, 0.717) than Model E + C (AUC 0.653; 95% CI 0.600, 0.706) based on cross-validated area-under-the-curve (Table 4). Monte Carlo cross-validation correction of the ROC curves for the logistic models demonstrates the overly optimistic prediction of the models when applied to the original sample (albeit with 20 multiple imputations for pain score) (Supplemental Fig. 2). Predictions for new persistent opioid use can be calculated based upon patient and geographic characteristics as described in the Supplemental Methods File using the estimated logistic regression coefficients from either model (Supplemental Table 4).

**Table 3:** Odds ratios (95% confidence intervals) for surgical patients developing persistent opioid use 90 to 180 days after surgery based on logistic regression analysis using 20 multiple imputations^a^ (*n* = 4,116)

Covariate	Model E + C	Model E + C + G
Male	1.01 (0.77, 1.33)	1.01 (0.77, 1.34)
Black^b^	1.33 (0.89, 2.00)	1.31 (0.87, 1.98)
Other race^b^	1.02 (0.59, 1.77)	1.06 (0.61, 1.84)
Age^c^	1.07 (1.00, 1.16)	1.08 (1.00, 1.16)*
Age-squared	1.00 (1.00, 1.00)	1.00 (1.00, 1.00)
Ever smoker	1.30 (0.99, 1.71)	1.26 (0.96, 1.65)
Pain Score^c^	1.11 (0.90, 1.36)	1.12 (0.90, 1.38)
Pain Score squared	1.00 (0.97, 1.02)	1.00 (0.97, 1.02)
Benzodiazepine Use	1.18 (0.82, 1.70)	1.18 (0.82, 1.71)
Back and neck pain	1.86 (1.42, 2.44)***	1.82 (1.39, 2.39)***
Joint disorders	1.57 (1.17, 2.10)**	1.58 (1.18, 2.11)**
Mood disorders	1.69 (1.27, 2.26)***	1.71 (1.28, 2.28)***
Unemployment rate^c^		1.10 (0.97, 1.26)
Unemployment rate squared		0.99 (0.99, 1.00)
Opioid retail prescriptions rate^c,d^		1.04 (1.00, 1.07)*
Opioid retail prescriptions rate squared		1.00 (1.00, 1.00)*
Drug poison death rate^c,e^		1.33 (1.09, 1.62)**
Drug poison death rate squared		0.99 (0.99, 1.00)**

^a^intercept estimate (standard error) is −5.76 (0.85) and −9.82 (1.25) for Model E + C (EHRs and claims), and Model E + C + G (EHRs, claims, and geographic factors) respectively. ^b^reference = white. ^c^With inclusion of quadratic effects, these are approximate odds ratios for an unit increase in the continuous variable. ^d^rate per 100 persons. ^e^estimated age-adjusted death rate per 100 persons.*p < 0.05; **p < 0.01; ***p < 0.001

**Table 4:** Average prediction performance of logistic regression models for new persistent opioid user (*n* = 4,116) based on 1,000 Monte-Carlo cross-validation samples. The 1st row cell entry for each model is the prediction performance statistic for predicting opioid use incidence calculated directly from the logistic model for incident opioid use based on 4,116 participants. The 2nd row cell entry is the prediction performance statistic, which is the average of 1,000 Monte-Carlo samples. The 3rd row cell entry is the Monte-Carlo cross-validation 95% CI for the prediction performance statistic

Model^a^	AUC	Brier score	Classification table with cutoff point^b^ of 0.06 for probability of opioid use			
			Sensitivity	Specificity	False positives	False negatives
E + C	0.676	0.054	58.7	65.0	90.4	3.9
	0.653	0.055	57.8	64.8	90.6	3.9
	(0.600, 0.706)	(0.047, 0.064)	(50.6, 64.2)	(59.1, 69.9)	(89.5, 91.7)	(3.4, 4.5)
E + C + G	0.698	0.054	62.1	65.5	89.8	3.5
	0.667	0.055	60.3	65.6	90.0	3.7
	(0.619, 0.717)	(0.047, 0.064)	(53.4, 66.3)	(60.5, 70.2)	(89.0, 91.1)	(3.2, 4.2)

^a^For both models, the 1st row cell entry is the average statistic across the 20 imputations; the 2nd and 3rd row cell entries are computed using a single imputation step for ordinal pain score for each Monte-Carlo sample replicate

^b^The cutoff point of 0.06 is determined by 245/4,116 = 0.06

With respect to the secondary question of whether the addition of claims data improved the ability to identify patients with relevant comorbidities and medication use, the addition of claims data more than doubled the frequency of patients with documented indications of comorbid medical conditions and benzodiazepine use based upon EHR alone (Supplemental Table 5). Moreover, while benzodiazepine use was positively associated with new persistent opioid use in both EHR alone (Supplemental Table 5) and EHR plus claims (Table 1) datasets, the association was statistically significant only in the combined dataset. In multivariable adjusted logistic models, the positive associations of all three co-morbid conditions were notably stronger in the combined dataset as was the overall prediction performance of Model E + C based on AUC (Supplemental Tables 6 and 7).

## Discussion

Approximately 6–10% of post-operative patients in nationwide claims data develop new persistent opioid use following surgery [2]. However, identification of these high-risk patients pre-operatively is currently obfuscated by variable and inconsistent risk factors across multiple studies [2, 7–9, 12] and a lack of pre-operative models to predict a patient’s risk of new persistent opioid use post-operatively. Further, variation in opioid prescribing practice in different communities may expose patients with similar personal risk factors to variable risk of persistent opioid use. To assess the impact of patient health factors and geographic factors on the development of persistent post-operative opioid use and how different data types contribute to identification of at-risk patients, this study utilized triplestore-driven data linkage methods to combine EHR, claims, and geographic data (CDC and ACS census-tract data) and developed models to identify contributing factors.

In agreement with previous work [2], this study identified that 6% of opioid naive surgical patients develop new persistent opioid use following surgery. Consistent with previous studies [2, 8, 9], we identified that benzodiazepine use, neck/back pain and mood disorders are predictive of persistent post-operative opioid use. Additional risk factors identified in this current study include joint disorders, residence in counties with high opioid retail prescriptions and residence in counties with high drug poison death rates. This analysis of naïve opioid surgical patients confirmed that co-morbid medical conditions are strong predictors of new persistent opioid use and further identified community level factors placing patients at higher risk. Therefore, this predictive model could be used to formulate a new persistent opioid use phenotype for use in clinical care, allowing for pre-operative identification of high-risk patients and optimization of both pre-operative counseling and alternative pain control strategies.

Addition of claims data strengthened the positive associations of each patient condition and the addition of geographic-area data did not significantly improve the predictive performance of the model based on boot-strapped area under the curve. While county level opioid retail prescription rates and drug poisoning rates published by the CDC were statistically significantly associated with new persistent opioid use after adjusting for co-morbidities and patient demographic factors, their added predictive value judged by prediction statistics such as cross-validated area-under-the-curve was modest and require larger samples to further evaluate. This remains promising for further study, particularly due to the identification of variables associated with social determinants of health (which are typically not captured in EHR and insurance data) in clinical research [30–34].

The improvement in our predictive model when utilizing both EHR and claims data demonstrated the importance of including claims data to improve upon the information available in the EHR. The addition of claims data does not provide a benefit to all EHR-driven analyses; for some instances, claims data has been shown to lead to poorer algorithmic performance [35, 36]. However, for this case, accurate and complete medication information was essential to our modeling effort. As an example, because of claims data’s rich medication dispensing information (as well as its reach beyond a single healthcare system’s data), the addition of claims data allowed us to identify 193 patients who developed new persistent opioid use in the cohort that would not have been identified using EHR data alone. Additionally, when claims data was included, additional patients with the co-morbid conditions or benzodiazepine use are greater than when claims are excluded. For example, based on EHR use only, benzodiazepine use is 4.5% (Supplemental Table 4), but when claims are included, it is 11.6% (Table 1). Similarly, for back and neck pain, joint disorders, and mood disorders, respectively, when claims are added to EHR data, prevalences increase from 10.6% to 28.6%, 21.6% to 54.7%, and 12.5% to 29.7%, respectively. Thus, even when its performance in our statistical models is set aside, it is fair to say that our computable phenotype for new persistent opioid user would have been substantially less powerful without claims data. Indeed, claims data and EHR data each have their own strengths; when used together in appropriate situations, each source can bring critical data points to research studies [37].

This study has several limitations, including a modest sample size, specific patient population identified through a private health insurance company, and a possible bias due to patients lost to follow-up. The first and foremost limitation was the modest sample size, which prohibited our ability to assess a greater number of community variables and the influence of patient factors with lower prevalence (< 10%) shown elsewhere to be associated with new opioid use [2]. Second, all patients had private health insurance and tended to live in geographic areas less impacted by unemployment, high opioid retail prescription rates, and opioid poison rates than the overall population, possibly limiting the generalizability of our findings. Third, as a retrospective study, data is limited by what is available in the EHR or claims data. Specifically, we lack patient-level data on unemployment, education level, income, and housing, which would have required patient questionnaires which were not feasible for this study design. Additionally, Charleson Comorbidity Index was previously identified as associated with new persistent post-operative opioid use [2], but is not routinely available in the CDWH and not available for inclusion our models. Finally, the 2016 CDC Guideline for Prescribing Opioids for Chronic Pain [38]was released during the study period and this may have impacted opioid prescribing or patient opioid use patterns.

Despite these limitations, a novelty of our study is the linkage of patient clinical factors from EHR with geographic factors from publicly available databases to develop a predictive model for new persistent opioid use following surgery. Future steps include expanding these predictive models to include additional patient populations, increasing patient numbers to allow for identification of high-risk patients pre-operatively, and developing clinical care pathways for high-risk patients. Effective pre-operative assessment of high-risk patients will allow for activation of additional resources, including improved counseling, implementation of alternative pain control strategies, and personalized follow-up approaches to reduce persistent post-operative opioid use.

## Conclusion

In this cohort study, predictors of persistent post-operative opioid use include a diagnosis of back and neck pain, joint disorders, mood disorders as well as the geographic factors of opioid retail prescription rate and drug death rates. The addition of geographic data to the traditional combination of EHR and claims data did not significantly improve identification of patients at high risk of persistent opioid use. Further studies with increased patient numbers are needed to substantiate and potentially expand these predictive models and develop clinical care pathways for high-risk patients.

## Supplementary Information

Supplementary Material 1

## Data Availability

The American Community Survey (ACS) and Centers for Disease Control and Prevention (CDC) are publicly available datasets. Access to electronic health records from the Carolina Data Warehouse for Health (CDWH) is limited by Health Insurance Portability and Accountability Act restrictions.

## Abbreviations

ACS: American Community Survey
AOR: Adjusted odds ratio
AUC: Area under the curve
CDC: Centers for Disease Control and Prevention
CDWH: Clinical Data Warehouse for Health
CI: Confidence interval
EHR: Electronic health record
ICD: International Classification of Diseases
RDF: Resource Description Framework
ROC: Receiver Operating Characteristic
