# Supplementary material for: Predictors of new persistent opioid use after surgery in adults
Source: Anesthesiol Perioper Sci. 2025 Jan 17;3(1):2. doi: 10.1007/s44254-024-00083-1 (PMC11880104; doi:10.1007/s44254-024-00083-1)
Supplement: Supplementary file 1 — Supplementary Material 1 [file 44254_2024_83_MOESM1_ESM.pdf]

Supplemental Information for

Predictors of New Persistent Opioid Use After Surgery in Adults

Journal: Anesthesiology and Perioperative Science

Kathryn H Gessner MD PhD[1], John S Preisser PhD[2,3], Emily Pfaff PhD MS[4], Rujin Wang PhD[2], Kellie Walters MPH[3], Robert Bradford BS[3], Marshall Clark BS[3], Mark Ehlers MD[1], Matthew Nielsen MD MS[1]

[1]*Department of Urology, University of North Carolina at Chapel Hill, Chapel Hill, NC*
[2]*Department of Biostatistics, Gillings School of Global Public Health, University of North Carolina, Chapel Hill, NC*
[3]*North Carolina Translational and Clinical Sciences Institute, University of North Carolina, Chapel Hill, NC*
[4]*Department of Medicine, University of North Carolina School of Medicine, Chapel Hill, NC*

Corresponding author:
Kathryn Hacker Gessner, MD, PhD
Email address: kathryn.gessner@unchealth.unc.edu
ORCID: 0000-0002-0335-4467

Supplemental Methods

Defining and extracting covariates from the EHR

We defined a patient-level smoking indicator variable as "never smoker" vs "ever smoker" from longitudinal data on multiple visits. To limit the amount of missing data, we did not impose a visit window on smoking status. Benzodiazepine use was defined based on the 12 month period prior to surgery excluding the index surgery date because their use may not indicate a predisposition for new persistent opioid use; instead, they are often used in the perioperative period as part of routine anesthesia protocols / premedications. Irregularly timed pain score data with a moderately high patient level of missingness (23%) was also accessed from the CDWH. Patient-rated pain was scored on a scale from 0 (least pain) to 9 (worst pain). A visit window was defined from surgery date (included) to 180 days post-surgery. We saved the mean pain score from the date that was closest to the index surgery date within the post-surgery window.

Preparation of Mental Health and Physical Pain Claims Dataset

To identify co-morbid medical conditions from EHR and claims data, a date window was defined as 1-year pre-surgery through the surgical date. Condition records were not included if assertion dates were not in the window. From condition identifiers (ICD-9 codes prior to October 10, 2015, and ICD-10 codes henceforward), we combined disorder types to create subcategories of the mental health diagnoses as previously described[5]: mood disorders (adjustment disorders, anxiety disorders and mood disorders), suicidality (suicide and self-harm), disruptive behavior disorders (attention deficit, conduct and disruptive disorders, impulse control disorders), personality disorders (personality disorders and schizophrenia), substance use disorders (substance use disorders and alcohol related disorders), and miscellaneous disorders (Table S1). Additionally, we used other condition identifiers to create indicator variables for physical pain, specifically for back/neck pain and joint disorders (Table S1).

There were 215,628 ICD9 and 320,908 ICD10 records for conditions in the triplestore data based on both EHR and claims data that included duplicate records for patients, visits and codes within patients (Figure S1). In these large datasets, there were 5,165 records with ICD9 codes and 9,180 records with ICD10 codes, respectively, that pertained to a set of *a priori* specified mental health conditions. Among these, 3300 records with ICD9 mental health codes and 5505 records with ICD10 mental health codes were from encounters within a visit window from one year prior to surgery through the index date of surgery. Deletion of duplicate records within patients resulted in 1018 patients with ICD9 codes for the mental health conditions and 1187 patients with ICD10 records for the mental health conditions. Merging datasets to remove matched (duplicate) ICD9 and ICD10 codes resulted in 1613 patients with one or more of the designated mental health conditions.

Using similar data manipulation methodology, there were 12,476 records with ICD9 codes and 20,569 records with ICD10 codes for "back and neck pain" and "joint disorder" (Figure S1). Among these, 8,503 ICD9 records and 12,706 ICD10 records were from encounters within a window from one year prior to surgery through the index date of surgery. Deletion of duplicate records within patients resulted in 1894 patients with ICD9 codes for one or more pain conditions and 1710 patients with ICD10 codes one or more pain conditions. Merging ICD9 and ICD10 codes to remove matched duplicate codes resulted in 2646 patients with "back and neck pain" or "joint disorder".

Imputation of missing data

Single imputation was used to impute missing data for race and "ever smoker." Specifically, conditional mode imputation was used for race with the most frequently occurring race category taken as the imputed value within each of 48 groups. These groups corresponded to the $2^4 \times 3=48$ cells in the multi-way contingency table formed by the cross-classifying gender (male or female), age group (under 60 years vs 60 years or older), ever smoker (yes or no), benzodiazepine use (yes or no) and number of comorbidities (three categories: 0, 1, versus 2 or more based on presence/absence of diagnosis codes for back and neck pain, joint disorder, mood disorder, respectively). Unconditional mode imputation was used to impute missing "ever smoker" status for the few patients missing this variable.

Multiple imputation was used to impute missing pain scores (for 946/4116 (23.0%) patients). An ordinal logistic regression imputation model based on cumulative logits and assuming proportional odds was fitted for pain (rounded up to the nearest integer when multiple scores were averaged) as a function of the patient-level factors from EHR and claims databases, specifically the main effects of gender, race, smoking status, benzodiazepine use, back and neck pain, joint disorders, mood disorders, suicidality, disruptive behavior disorders, personality disorders

and schizophrenia, substance use disorders, and miscellaneous disorders. The estimated effects in the models described above based on 20 imputations were computed using SAS Proc Logistic and MIANALYZE v 9.4 (SAS, Cary, NC, USA).

Prediction Equations for new persistent opioid use

Predictions for new persistent opioid use for opioid-naïve surgical patients can be calculated based upon patient and geographic characteristics using the estimated logistic regression coefficients from a prediction model. Specifically, the probability that a patient becomes a new persistent opioid user is calculated as $p = \exp(lp)\,/[1 + \exp(lp)]$ with the linear predictor lp defined by the regression coefficients from the model of choice. For the model E+C, $lp = \beta_0 + \sum_{j=1}^{12} x_j\beta_j$, where the twelve covariates, $x_1, x_2, \ldots, x_{12}$ correspond to Male, Black,…, Mood disorders, respectively. Based on the estimated regression coefficients for Model E+C from Supplemental Table 4,

$$\widehat{lp} = -5.757 + 0.011x_1 + 0.288x_2 + 0.019x_3 + 0.071x_4 - 0.00064x_5 + 0.262x_6 + 0.101x_7 - 0.0018x_8 + 0.165x_9 + 0.619x_{10} + 0.452x_{11} + 0.526x_{12}$$

where $x_5 = x_4^2$ and $x_8 = x_7^2$ are quadratic effects for age and pain score, respectively. As an illustration, consider two 45 year old white women, both with a pain score of 4, and without Joint or Mood disorders; in statistical notation, $x_1 = x_2 = x_3 = x_{11} = x_{12} = 0;\ x_4 = 45;\ x_7 = 4;$ Now, the first woman has never been a smoker ($x_6 = 0$), has no benzodiazepine use ($x_9 = 0$), and no back/neck pain ($x_{10} = 0$). By the above equation, this woman has a predicted probability of 0.03 of new persistent opioid use. Next, suppose, the second woman is a smoker ($x_6 = 1$), has used benzodiazepines ($x_9 = 1$), and has back/neck pain ($x_{10} = 1$) documented in the EHR and/or claims. By the prediction equation for Model E+C, this second woman has a predicted probability of 0.08 of new persistent opioid use, or more than twice the risk as the first woman. Similar calculations can be performed for individuals with different constellations of risk factors including, potentially, geographic factors using the estimated prediction equation regression coefficients from Model E+C+G in Supplemental Table 4.

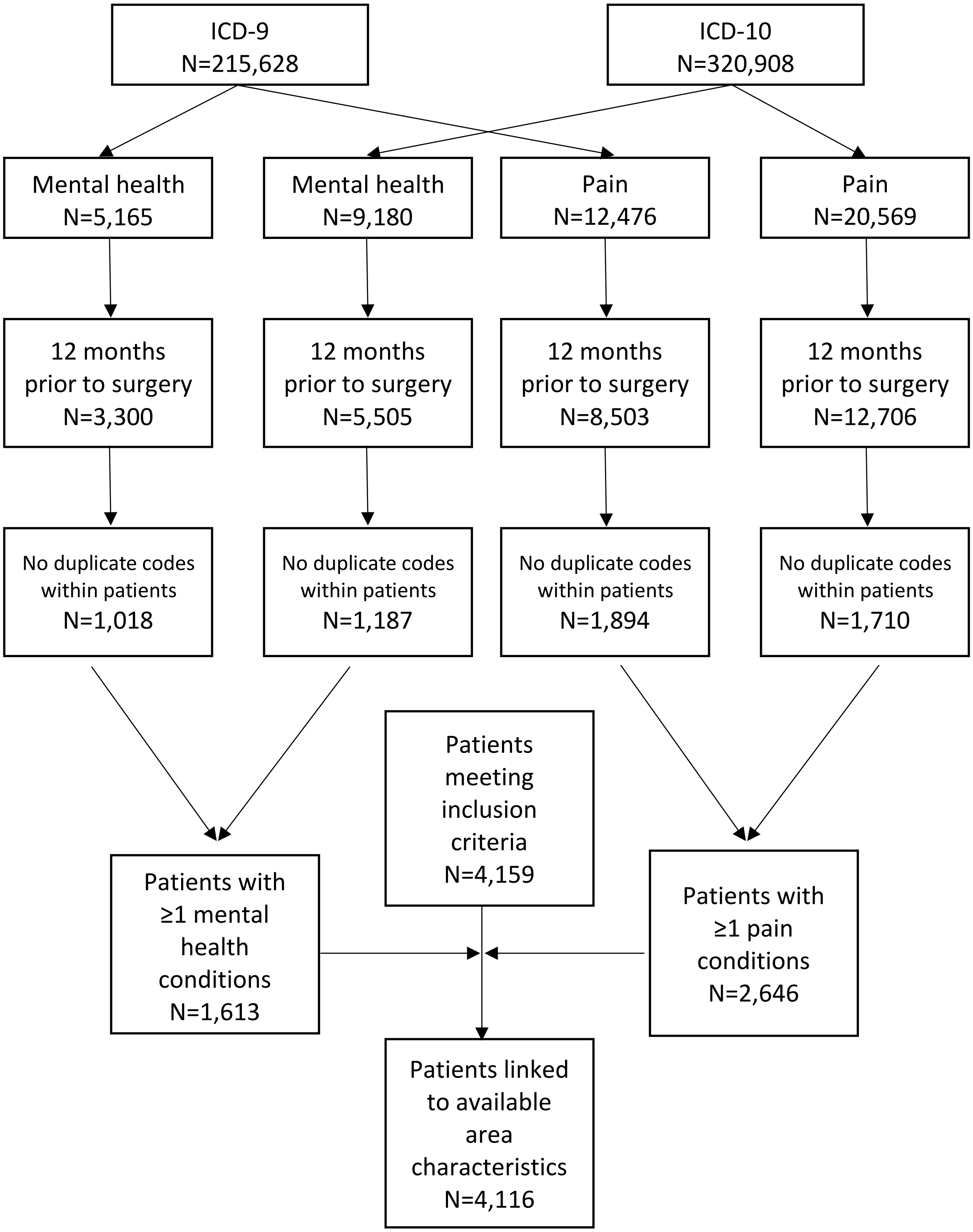

Supplemental Figure 1. Construction of the mental health and physical pain dataset of 4,116 opioid-naïve surgery patients based on EHR and claims data for prediction modeling of new persistent opioid use.

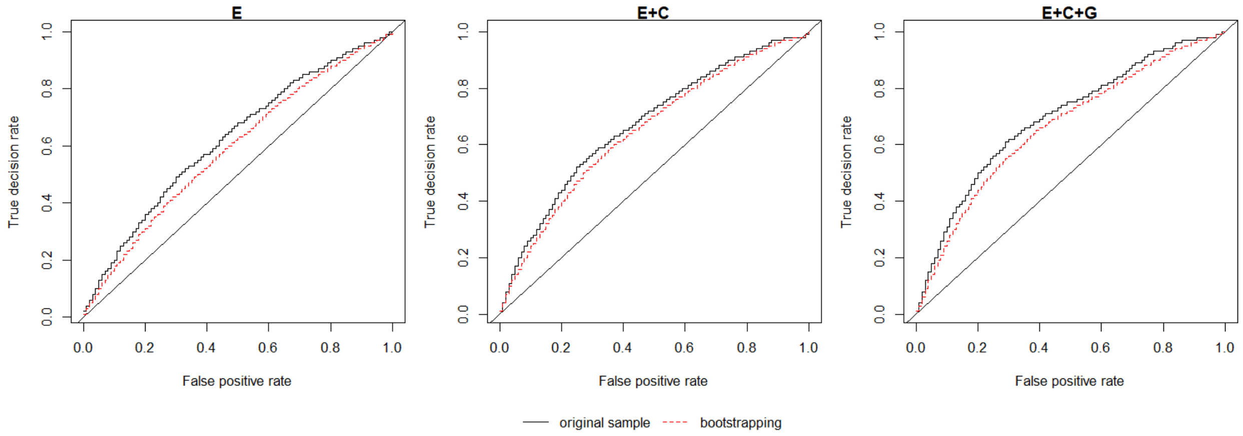

Supplemental Figure 2. ROC curves for prediction models showing the overly optimistic prediction based on a single model versus Monte Carlo cross-validation.

Supplemental Table 1. ICD-9 and ICD-10 codes for mental health and pain diagnoses.

Condition	ICD-9	ICD-10
Adjustment disorder	309.0, 309.1, 309.22, 309.23, 309.24, 309.28, 309.29, 309.3, 309.4, 309.82, 309.83, 309.89, 309.9	F4320.X, F4321.X, F4322.X, F4323.X, F4324.X, F4325.X, F4329.X, F438.X, F439.X
Anxiety disorder	293.84, 300.00, 300.01, 300.02, 300.09, 300.10, 300.20, 300.21, 300.22, 300.23, 300.29, 300.3, 300.5, 300.89, 300.9, 308.0, 308.1, 308.2, 308.3, 308.4, 308.9, 309.81, 313.0, 313.1, 313.21, 313.22, 313.3, 313.82, 313.83	F064.X, F4000.X, F4001.X, F4002.X, F4010.X, F4011.X, F40210.X, F40218.X, F40220.X, F40228.X, F40230.X, F40231.X, F40232.X, F40233.X, F40240.X, F40241.X, F40242.X, F40243.X, F40248.X, F40290.X, F40291.X, F40298.X, F408.X, F409.X, F410.X, F411.X, F413.X, F418.X, F419.X, F42.X, F422.X, F423.X, F424.X, F428.X, F429.X, F430.X, F4310.X, F4311.X, F4312.X, F488.X, F489.X, R452.X, R453.X, R454.X, R455.X, R456.X, R457.X, R4581.X, R4582.X, R4583.X, R4584.X
Attention deficit, conduct disorders	312.00, 312.01, 312.02, 312.03, 312.10, 312.11, 312.12, 312.13, 312.20, 312.21, 312.22, 312.23, 312.4, 312.8, 312.81, 312.82, 312.89, 312.9, 313.81, 314.00, 314.01, 314.1, 314.2, 314.8, 314.9	F900.X, F901.X, F902.X, F908.X, F909.X, F910.X, F911.X, F912.X, F913.X, F918.X, F919.X, R460.X, R461.X, R462.X, R463.X, R464.X, R465.X, R466.X, R467.X, R4681.X, R4689.X
Impulse control disorders	312.30, 312.31, 312.32, 312.33, 312.34, 312.35, 312.39	F630.X, F631.X, F632.X, F633.X, F6381.X, F6389.X, F639.X, R45850.X
Mood disorders	293.83, 296.00, 296.01, 296.02, 296.03, 296.04, 296.05, 296.06, 296.10, 296.11, 296.12, 296.13, 296.14, 296.15, 296.16, 296.20, 296.21, 296.22, 296.23, 296.24, 296.25, 296.26, 296.30, 296.31, 296.32, 296.33, 296.34, 296.35, 296.36, 296.40, 296.41, 296.42, 296.43, 296.44, 296.45, 296.46, 296.50, 296.51, 296.52, 296.53, 296.54, 296.55, 296.56, 296.60, 296.61, 296.62, 296.63, 296.64, 296.65, 296.66, 296.7, 296.80, 296.81, 296.82, 296.89, 296.90, 296.99, 300.4, 311	F0630.X, F0631.X, F0632.X, F0633.X, F0634.X, F3010.X, F3011.X, F3012.X, F3013.X, F302.X, F303.X, F304.X, F308.X, F309.X, F310.X, F3110.X, F3111.X, F3112.X, F3113.X, F312.X, F3130.X, F3131.X, F3132.X, F314.X, F315.X, F3160.X, F3161.X, F3162.X, F3163.X, F3164.X, F3170.X, F3171.X, F3172.X, F3173.X, F3174.X, F3175.X, F3176.X, F3177.X, F3178.X, F3181.X, F3189.X, F319.X, F320.X, F321.X, F322.X, F323.X, F324.X, F325.X, F328.X, F3281.X, F3289.X, F329.X, F330.X, F331.X, F332.X, F333.X, F3340.X, F3341.X, F3342.X, F338.X, F339.X, F340.X, F341.X, F348.X, F3481.X, F3489.X, F349.X, F39.X, R4586.X
Personality disorders	301.0, 301.10, 301.11, 301.12, 301.13, 301.20, 301.21, 301.22, 301.3, 301.4, 301.50, 301.51, 301.59, 301.6, 301.7, 301.81, 301.82, 301.83, 301.84, 301.89, 301.9	F600.X, F601.X, F602.X, F603.X, F604.X, F605.X, F606.X, F607.X, F6081.X, F6089.X, F609.X, F69.X
Schizophrenia	293.81, 293.82, 295.00, 295.01, 295.02, 295.03, 295.04, 295.05, 295.10, 295.11, 295.12, 295.13, 295.14, 295.15, 295.20, 295.21, 295.22, 295.23, 295.24, 295.25, 295.30, 295.31, 295.32, 295.33, 295.34, 295.35, 295.40, 295.41, 295.42, 295.43, 295.44, 295.45, 295.50, 295.51, 295.52, 295.53,	F060.X, F062.X, F200.X, F201.X, F202.X, F203.X, F205.X, F2081.X, F2089.X, F209.X, F21.X, F22.X, F23.X, F24.X, F250.X, F251.X, F258.X, F259.X, F28.X, F29.X

	295.54, 295.55, 295.60, 295.61, 295.62, 295.63, 295.64, 295.65, 295.70, 295.71, 295.72, 295.73, 295.74, 295.75, 295.80, 295.81, 295.82, 295.83, 295.84, 295.85, 295.90, 295.91, 295.92, 295.93, 295.94, 295.95, 297.0, 297.1, 297.2, 297.3, 297.8, 297.9, 298.0, 298.1, 298.2, 298.3, 298.4, 298.8, 298.9	
Alcohol related disorders	291.0, 291.1, 291.2, 291.3, 291.4, 291.5, 291.8, 291.81, 291.82, 291.89, 291.9, 303.00, 303.01, 303.02, 303.03, 303.90, 303.91, 303.92, 303.93, 305.00, 305.01, 305.02, 305.03, 357.5, 425.5, 535.3, 535.30, 535.31, 571.0, 571.1, 571.2, 571.3, 760.71, 980.0	F1010.X, F1011.X, F10120.X, F10121.X, F10129.X, F1014.X, F10150.X, F10151.X, F10159.X, F10180.X, F10181.X, F10182.X, F10188.X, F1019.X, F1020.X, F1021.X, F10220.X, F10221.X, F10229.X, F10230.X, F10231.X, F10232.X, F10239.X, F1024.X, F10250.X, F10251.X, F10259.X, F1026.X, F1027.X, F10280.X, F10281.X, F10282.X, F10288.X, F1029.X, F10920.X, F10921.X, F10929.X, F1094.X, F10950.X, F10951.X, F10959.X, F1096.X, F1097.X, F10980.X, F10981.X, F10982.X, F10988.X, F1099.X, G621.X, I426.X, K2920.X, K2921.X, K700.X, K7010.X, K7011.X, K702.X, K7030.X, K7031.X, K7040.X, K709.X, O99310.X, O99311.X, O99312.X, O99313.X, O99314.X, O99315.X, P043.X, Q860.X

Substance related disorders	292.0, 292.11, 292.12, 292.2, 292.81, 292.82, 292.83, 292.84, 292.85, 292.89, 292.9, 304.00, 304.01, 304.02, 304.03, 304.10, 304.11, 304.12, 304.13, 304.20, 304.21, 304.22, 304.23, 304.30, 304.31, 304.32, 304.33, 304.40, 304.41, 304.42, 304.43, 304.50, 304.51, 304.52, 304.53, 304.60, 304.61, 304.62, 304.63, 304.70, 304.71, 304.72, 304.73, 304.80, 304.81, 304.82, 304.83, 304.90, 304.91, 304.92, 304.93, 305.20, 305.21, 305.22, 305.23, 305.30, 305.31, 305.32, 305.33, 305.40, 305.41, 305.42, 305.43, 305.50, 305.51, 305.52, 305.53, 305.60, 305.61, 305.62, 305.63, 305.70, 305.71, 305.72, 305.73, 305.80, 305.81, 305.82, 305.83, 305.90, 305.91, 305.92, 305.93, 648.30, 648.31, 648.32, 648.33, 648.34, 655.50, 655.51, 655.53, 760.72, 760.73, 760.75, 779.5, 965.00, 965.01, 965.02, 965.09, V65.42	F1110.X, F1111.X, F11120.X, F11121.X, F11122.X, F11129.X, F1114.X, F11150.X, F11151.X, F11159.X, F11181.X, F11182.X, F11188.X, F1119.X, F1120.X, F1121.X, F11220.X, F11221.X, F11222.X, F11229.X, F1123.X, F1124.X, F11250.X, F11251.X, F11259.X, F11281.X, F11282.X, F11288.X, F1129.X, F1190.X, F11920.X, F11921.X, F11922.X, F11929.X, F1193.X, F1194.X, F11950.X, F11951.X, F11959.X, F11981.X, F11982.X, F11988.X, F1199.X, F1210.X, F1211.X, F12120.X, F12121.X, F12122.X, F12129.X, F12150.X, F12151.X, F12159.X, F12180.X, F12188.X, F1219.X, F1220.X, F1221.X, F12220.X, F12221.X, F12222.X, F12229.X, F1223.X, F12250.X, F12251.X, F12259.X, F12280.X, F12288.X, F1229.X, F1290.X, F12920.X, F12921.X, F12922.X, F12929.X, F1293.X, F12950.X, F12951.X, F12959.X, F12980.X, F12988.X, F1299.X, F1310.X, F1311.X, F13120.X, F13121.X, F13129.X, F1314.X, F13150.X, F13151.X, F13159.X, F13180.X, F13181.X, F13182.X, F13188.X, F1319.X, F1320.X, F1321.X, F13220.X, F13221.X, F13229.X, F13230.X, F13231.X, F13232.X, F13239.X, F1324.X, F13250.X, F13251.X, F13259.X, F1326.X, F1327.X, F13280.X, F13281.X, F13282.X, F13288.X, F1329.X, F1390.X, F13920.X, F13921.X, F13929.X, F13930.X, F13931.X, F13932.X, F13939.X, F1394.X, F13950.X, F13951.X, F13959.X, F1396.X, F1397.X, F13980.X, F13981.X, F13982.X, F13988.X, F1399.X, F1410.X, F1411.X, F14120.X, F14121.X, F14122.X, F14129.X, F1414.X, F14150.X, F14151.X, F14159.X, F14180.X, F14181.X, F14182.X, F14188.X, F1419.X, F1420.X, F1421.X, F14220.X, F14221.X, F14222.X, F14229.X, F1423.X, F1424.X, F14250.X, F14251.X, F14259.X, F14280.X, F14281.X, F14282.X, F14288.X, F1429.X, F1490.X, F14920.X, F14921.X, F14922.X, F14929.X, F1494.X, F14950.X, F14951.X, F14959.X, F14980.X, F14981.X, F14982.X, F14988.X, F1499.X, F1510.X, F1511.X, F15120.X, F15121.X, F15122.X, F15129.X, F1514.X, F15150.X, F15151.X, F15159.X, F15180.X, F15181.X, F15182.X, F15188.X, F1519.X, F1520.X, F1521.X, F15220.X, F15221.X, F15222.X, F15229.X, F1523.X, F1524.X, F15250.X, F15251.X, F15259.X, F15280.X, F15281.X, F15282.X, F15288.X, F1529.X, F1590.X, F15920.X, F15921.X, F15922.X, F15929.X, F1593.X, F1594.X, F15950.X, F15951.X, F15959.X, F15980.X, F15981.X, F15982.X, F15988.X, F1599.X, F1610.X, F1611.X, F16120.X, F16121.X, F16122.X, F16129.X, F1614.X, F16150.X, F16151.X, F16159.X, F16180.X, F16183.X, F16188.X, F1619.X, F1620.X, F1621.X, F16220.X, F16221.X, F16229.X, F1624.X, F16250.X, F16251.X, F16259.X, F16280.X, F16283.X, F16288.X, F1629.X, F1690.X, F16920.X, F16921.X, F16929.X, F1694.X, F16950.X, F16951.X, F16959.X, F16980.X, F16983.X, F16988.X, F1699.X, F17200.X, F17201.X, F17203.X, F17208.X, F17209.X, F17210.X, F17211.X, F17213.X, F17218.X, F17219.X, F17220.X, F17221.X, F17223.X, F17228.X, F17229.X, F17290.X, F17291.X,

		F17293.X, F17298.X, F17299.X, F1810.X, F1811.X, F18120.X, F18121.X, F18129.X, F1814.X, F18150.X, F18151.X, F18159.X, F1817.X, F18180.X, F18188.X, F1819.X, F1820.X, F1821.X, F18220.X, F18221.X, F18229.X, F1824.X, F18250.X, F18251.X, F18259.X, F1827.X, F18280.X, F18288.X, F1829.X, F1890.X, F18920.X, F18921.X, F18929.X, F1894.X, F18950.X, F18951.X, F18959.X, F1897.X, F18980.X, F18988.X, F1899.X, F1910.X, F1911.X, F19120.X, F19121.X, F19122.X, F19129.X, F1914.X, F19150.X, F19151.X, F19159.X, F1916.X, F1917.X, F19180.X, F19181.X, F19182.X, F19188.X, F1919.X, F1920.X, F1921.X, F19220.X, F19221.X, F19222.X, F19229.X, F19230.X, F19231.X, F19232.X, F19239.X, F1924.X, F19250.X, F19251.X, F19259.X, F1926.X, F1927.X, F19280.X, F19281.X, F19282.X, F19288.X, F1929.X, F1990.X, F19920.X, F19921.X, F19922.X, F19929.X, F19930.X, F19931.X, F19932.X, F19939.X, F1994.X, F19950.X, F19951.X, F19959.X, F1996.X, F1997.X, F19980.X, F19981.X, F19982.X, F19988.X, F1999.X, F550.X, F551.X, F552.X, F553.X, F554.X, F558.X, O355XX0.X, O355XX1.X, O355XX2.X, O355XX3.X, O355XX4.X, O355XX5.X, O355XX9.X, O99320.X, O99321.X, O99322.X, O99323.X, O99324.X, O99325.X, P0441.X, P0449.X, P961.X, P962.X, T400X1A.X, T400X1D.X, T400X1S.X, T400X3A.X, T400X3D.X, T400X3S.X, T400X4A.X, T400X4D.X, T400X4S.X, T400X5A.X, T400X5D.X, T400X5S.X, T400X6A.X, T400X6D.X, T400X6S.X, T401X1A.X, T401X1D.X, T401X1S.X, T401X3A.X, T401X3D.X, T401X3S.X, T401X4A.X, T401X4D.X, T401X4S.X, T401X5A.X, T401X5D.X, T401X5S.X, T405X1A.X, T405X1D.X, T405X1S.X, T405X3A.X, T405X3D.X, T405X3S.X, T405X4A.X, T405X4D.X, T405X4S.X, T405X5A.X, T405X5D.X, T405X5S.X, T405X6A.X, T405X6D.X, T405X6S.X, T407X1A.X, T407X1D.X, T407X1S.X, T407X3A.X, T407X3D.X, T407X3S.X, T407X4A.X, T407X4D.X, T407X4S.X, T407X5A.X, T407X5D.X, T407X5S.X, T407X6A.X, T407X6D.X, T407X6S.X, T408X1A.X, T408X1D.X, T408X1S.X, T408X3A.X, T408X3D.X, T408X3S.X, T408X4A.X, T408X4D.X, T408X4S.X, T408X5A.X, T408X5D.X, T408X5S.X, T40901A.X, T40901D.X, T40901S.X, T40903A.X, T40903D.X, T40903S.X, T40904A.X, T40904D.X, T40904S.X, T40905A.X, T40905D.X, T40905S.X, T40906A.X, T40906D.X, T40906S.X, T40991A.X, T40991D.X, T40991S.X, T40993A.X, T40993D.X, T40993S.X, T40994A.X, T40994D.X, T40994S.X, T40995A.X, T40995D.X, T40995S.X, T40996A.X, T40996D.X, T40996S.X

Suicide and self harm	E95.00, E95.01, E95.02, E95.03, E95.04, E95.05, E95.06, E95.07, E95.08, E95.09, E95.10, E95.11, E95.18, E95.20, E95.21, E95.28, E95.29, E95.30, E95.31, E95.38, E95.39, E95.4, E95.50, E95.51, E95.52, E95.53, E95.54, E95.55, E95.56, E95.57, E95.59, E95.6, E95.70, E95.71, E95.72, E95.79, E95.80, E95.81, E95.82, E95.83, E95.84, E95.85, E95.86, E95.87, E95.88, E95.89, E95.9, V62.84	R45851.X, T1491.X, T1491XA.X, T1491XD.X, T1491XS.X, T360X2A.X, T360X2D.X, T360X2S.X, T361X2A.X, T361X2D.X, T361X2S.X, T362X2A.X, T362X2D.X, T362X2S.X, T363X2A.X, T363X2D.X, T363X2S.X, T364X2A.X, T364X2D.X, T364X2S.X, T365X2A.X, T365X2D.X, T365X2S.X, T366X2A.X, T366X2D.X, T366X2S.X, T367X2A.X, T367X2D.X, T367X2S.X, T368X2A.X, T368X2D.X, T368X2S.X, T3692XA.X, T3692XD.X, T3692XS.X, T370X2A.X, T370X2D.X, T370X2S.X, T371X2A.X, T371X2D.X, T371X2S.X, T372X2A.X, T372X2D.X, T372X2S.X, T373X2A.X, T373X2D.X, T373X2S.X, T374X2A.X, T374X2D.X, T374X2S.X, T375X2A.X, T375X2D.X, T375X2S.X, T378X2A.X, T378X2D.X, T378X2S.X, T3792XA.X, T3792XD.X, T3792XS.X, T380X2A.X, T380X2D.X, T380X2S.X, T381X2A.X, T381X2D.X, T381X2S.X, T382X2A.X, T382X2D.X, T382X2S.X, T383X2A.X, T383X2D.X, T383X2S.X, T384X2A.X, T384X2D.X, T384X2S.X, T385X2A.X, T385X2D.X, T385X2S.X, T386X2A.X, T386X2D.X, T386X2S.X, T387X2A.X, T387X2D.X, T387X2S.X, T38802A.X, T38802D.X, T38802S.X, T38812A.X, T38812D.X, T38812S.X, T38892A.X, T38892D.X, T38892S.X, T38902A.X, T38902D.X, T38902S.X, T38992A.X, T38992D.X, T38992S.X, T39012A.X, T39012D.X, T39012S.X, T39092A.X, T39092D.X, T39092S.X, T391X2A.X, T391X2D.X, T391X2S.X, T392X2A.X, T392X2D.X, T392X2S.X, T39312A.X, T39312D.X, T39312S.X, T39392A.X, T39392D.X, T39392S.X, T394X2A.X, T394X2D.X, T394X2S.X, T398X2A.X, T398X2D.X, T398X2S.X, T3992XA.X, T3992XD.X, T3992XS.X, T400X2A.X, T400X2D.X, T400X2S.X, T401X2A.X, T401X2D.X, T401X2S.X, T402X2A.X, T402X2D.X, T402X2S.X, T403X2A.X, T403X2D.X, T403X2S.X, T404X2A.X, T404X2D.X, T404X2S.X, T405X2A.X, T405X2D.X, T405X2S.X, T40602A.X, T40602D.X, T40602S.X, T40692A.X, T40692D.X, T40692S.X, T407X2A.X, T407X2D.X, T407X2S.X, T408X2A.X, T408X2D.X, T408X2S.X, T40902A.X, T40902D.X, T40902S.X, T40992A.X, T40992D.X, T40992S.X, T410X2A.X, T410X2D.X, T410X2S.X, T411X2A.X, T411X2D.X, T411X2S.X, T41202A.X, T41202D.X, T41202S.X, T41292A.X, T41292D.X, T41292S.X, T413X2A.X, T413X2D.X, T413X2S.X, T4142XA.X, T4142XD.X, T4142XS.X, T415X2A.X, T415X2D.X, T415X2S.X, T420X2A.X, T420X2D.X, T420X2S.X, T421X2A.X, T421X2D.X, T421X2S.X, T422X2A.X, T422X2D.X, T422X2S.X, T423X2A.X, T423X2D.X, T423X2S.X, T424X2A.X, T424X2D.X, T424X2S.X, T425X2A.X, T425X2D.X, T425X2S.X, T426X2A.X, T426X2D.X, T426X2S.X, T4272XA.X, T4272XD.X, T4272XS.X, T428X2A.X, T428X2D.X, T428X2S.X, T43012A.X, T43012D.X, T43012S.X, T43022A.X, T43022D.X, T43022S.X, T431X2A.X, T431X2D.X, T431X2S.X, T43202A.X, T43202D.X, T43202S.X, T43212A.X, T43212D.X, T43212S.X,

		T43222A.X, T43222D.X, T43222S.X, T43292A.X, T43292D.X, T43292S.X, T433X2A.X, T433X2D.X, T433X2S.X, T434X2A.X, T434X2D.X, T434X2S.X, T43502A.X, T43502D.X, T43502S.X, T43592A.X, T43592D.X, T43592S.X, T43602A.X, T43602D.X, T43602S.X, T43612A.X, T43612D.X, T43612S.X, T43622A.X, T43622D.X, T43622S.X, T43632A.X, T43632D.X, T43632S.X, T43692A.X, T43692D.X, T43692S.X, T438X2A.X, T438X2D.X, T438X2S.X, T4392XA.X, T4392XD.X, T4392XS.X, T440X2A.X, T440X2D.X, T440X2S.X, T441X2A.X, T441X2D.X, T441X2S.X, T442X2A.X, T442X2D.X, T442X2S.X, T443X2A.X, T443X2D.X, T443X2S.X, T444X2A.X, T444X2D.X, T444X2S.X, T445X2A.X, T445X2D.X, T445X2S.X, T446X2A.X, T446X2D.X, T446X2S.X, T447X2A.X, T447X2D.X, T447X2S.X, T448X2A.X, T448X2D.X, T448X2S.X, T44902A.X, T44902D.X, T44902S.X, T44992A.X, T44992D.X, T44992S.X, T450X2A.X, T450X2D.X, T450X2S.X, T451X2A.X, T451X2D.X, T451X2S.X, T452X2A.X, T452X2D.X, T452X2S.X, T453X2A.X, T453X2D.X, T453X2S.X, T454X2A.X, T454X2D.X, T454X2S.X, T45512A.X, T45512D.X, T45512S.X, T45522A.X, T45522D.X, T45522S.X, T45602A.X, T45602D.X, T45602S.X, T45612A.X, T45612D.X, T45612S.X, T45622A.X, T45622D.X, T45622S.X, T45692A.X, T45692D.X, T45692S.X, T457X2A.X, T457X2D.X, T457X2S.X, T458X2A.X, T458X2D.X, T458X2S.X, T4592XA.X, T4592XD.X, T4592XS.X, T460X2A.X, T460X2D.X, T460X2S.X, T461X2A.X, T461X2D.X, T461X2S.X, T462X2A.X, T462X2D.X, T462X2S.X, T463X2A.X, T463X2D.X, T463X2S.X, T464X2A.X, T464X2D.X, T464X2S.X, T465X2A.X, T465X2D.X, T465X2S.X, T466X2A.X, T466X2D.X, T466X2S.X, T467X2A.X, T467X2D.X, T467X2S.X, T468X2A.X, T468X2D.X, T468X2S.X, T46902A.X, T46902D.X, T46902S.X, T46992A.X, T46992D.X, T46992S.X, T470X2A.X, T470X2D.X, T470X2S.X, T471X2A.X, T471X2D.X, T471X2S.X, T472X2A.X, T472X2D.X, T472X2S.X, T473X2A.X, T473X2D.X, T473X2S.X, T474X2A.X, T474X2D.X, T474X2S.X, T475X2A.X, T475X2D.X, T475X2S.X, T476X2A.X, T476X2D.X, T476X2S.X, T477X2A.X, T477X2D.X, T477X2S.X, T478X2A.X, T478X2D.X, T478X2S.X, T4792XA.X, T4792XD.X, T4792XS.X, T480X2A.X, T480X2D.X, T480X2S.X, T481X2A.X, T481X2D.X, T481X2S.X, T48202A.X, T48202D.X, T48202S.X, T48292A.X, T48292D.X, T48292S.X, T483X2A.X, T483X2D.X, T483X2S.X, T484X2A.X, T484X2D.X, T484X2S.X, T485X2A.X, T485X2D.X, T485X2S.X, T486X2A.X, T486X2D.X, T486X2S.X, T48902A.X, T48902D.X, T48902S.X, T48992A.X, T48992D.X, T48992S.X, T490X2A.X, T490X2D.X, T490X2S.X, T491X2A.X, T491X2D.X, T491X2S.X, T492X2A.X, T492X2D.X, T492X2S.X, T493X2A.X, T493X2D.X, T493X2S.X, T494X2A.X, T494X2D.X, T494X2S.X, T495X2A.X, T495X2D.X,

		T495X2S.X, T496X2A.X, T496X2D.X, T496X2S.X, T497X2A.X, T497X2D.X, T497X2S.X, T498X2A.X, T498X2D.X, T498X2S.X, T4992XA.X, T4992XD.X, T4992XS.X, T500X2A.X, T500X2D.X, T500X2S.X, T501X2A.X, T501X2D.X, T501X2S.X, T502X2A.X, T502X2D.X, T502X2S.X, T503X2A.X, T503X2D.X, T503X2S.X, T504X2A.X, T504X2D.X, T504X2S.X, T505X2A.X, T505X2D.X, T505X2S.X, T506X2A.X, T506X2D.X, T506X2S.X, T507X2A.X, T507X2D.X, T507X2S.X, T508X2A.X, T508X2D.X, T508X2S.X, T50902A.X, T50902D.X, T50902S.X, T50992A.X, T50992D.X, T50992S.X, T50A12A.X, T50A12D.X, T50A12S.X, T50A22A.X, T50A22D.X, T50A22S.X, T50A92A.X, T50A92D.X, T50A92S.X, T50B12A.X, T50B12D.X, T50B12S.X, T50B92A.X, T50B92D.X, T50B92S.X, T50Z12A.X, T50Z12D.X, T50Z12S.X, T50Z92A.X, T50Z92D.X, T50Z92S.X, T510X2A.X, T510X2D.X, T510X2S.X, T511X2A.X, T511X2D.X, T511X2S.X, T512X2A.X, T512X2D.X, T512X2S.X, T513X2A.X, T513X2D.X, T513X2S.X, T518X2A.X, T518X2D.X, T518X2S.X, T5192XA.X, T5192XD.X, T5192XS.X, T520X2A.X, T520X2D.X, T520X2S.X, T521X2A.X, T521X2D.X, T521X2S.X, T522X2A.X, T522X2D.X, T522X2S.X, T523X2A.X, T523X2D.X, T523X2S.X, T524X2A.X, T524X2D.X, T524X2S.X, T528X2A.X, T528X2D.X, T528X2S.X, T5292XA.X, T5292XD.X, T5292XS.X, T530X2A.X, T530X2D.X, T530X2S.X, T531X2A.X, T531X2D.X, T531X2S.X, T532X2A.X, T532X2D.X, T532X2S.X, T533X2A.X, T533X2D.X, T533X2S.X, T534X2A.X, T534X2D.X, T534X2S.X, T535X2A.X, T535X2D.X, T535X2S.X, T536X2A.X, T536X2D.X, T536X2S.X, T537X2A.X, T537X2D.X, T537X2S.X, T5392XA.X, T5392XD.X, T5392XS.X, T540X2A.X, T540X2D.X, T540X2S.X, T541X2A.X, T541X2D.X, T541X2S.X, T542X2A.X, T542X2D.X, T542X2S.X, T543X2A.X, T543X2D.X, T543X2S.X, T5492XA.X, T5492XD.X, T5492XS.X, T550X2A.X, T550X2D.X, T550X2S.X, T551X2A.X, T551X2D.X, T551X2S.X, T560X2A.X, T560X2D.X, T560X2S.X, T561X2A.X, T561X2D.X, T561X2S.X, T562X2A.X, T562X2D.X, T562X2S.X, T563X2A.X, T563X2D.X, T563X2S.X, T564X2A.X, T564X2D.X, T564X2S.X, T565X2A.X, T565X2D.X, T565X2S.X, T566X2A.X, T566X2D.X, T566X2S.X, T567X2A.X, T567X2D.X, T567X2S.X, T56812A.X, T56812D.X, T56812S.X, T56892A.X, T56892D.X, T56892S.X, T5692XA.X, T5692XD.X, T5692XS.X, T570X2A.X, T570X2D.X, T570X2S.X, T571X2A.X, T571X2D.X, T571X2S.X, T572X2A.X, T572X2D.X, T572X2S.X, T573X2A.X, T573X2D.X, T573X2S.X, T578X2A.X, T578X2D.X, T578X2S.X, T5792XA.X, T5792XD.X, T5792XS.X, T5802XA.X, T5802XD.X, T5802XS.X, T5812XA.X, T5812XD.X, T5812XS.X, T582X2A.X, T582X2D.X, T582X2S.X, T588X2A.X, T588X2D.X, T588X2S.X, T5892XA.X, T5892XD.X, T5892XS.X, T590X2A.X, T590X2D.X, T590X2S.X, T591X2A.X,

		T591X2D.X, T591X2S.X, T592X2A.X, T592X2D.X, T592X2S.X, T593X2A.X, T593X2D.X, T593X2S.X, T594X2A.X, T594X2D.X, T594X2S.X, T595X2A.X, T595X2D.X, T595X2S.X, T596X2A.X, T596X2D.X, T596X2S.X, T597X2A.X, T597X2D.X, T597X2S.X, T59812A.X, T59812D.X, T59812S.X, T59892A.X, T59892D.X, T59892S.X, T5992XA.X, T5992XD.X, T5992XS.X, T600X2A.X, T600X2D.X, T600X2S.X, T601X2A.X, T601X2D.X, T601X2S.X, T602X2A.X, T602X2D.X, T602X2S.X, T603X2A.X, T603X2D.X, T603X2S.X, T604X2A.X, T604X2D.X, T604X2S.X, T608X2A.X, T608X2D.X, T608X2S.X, T6092XA.X, T6092XD.X, T6092XS.X, T6102XA.X, T6102XD.X, T6102XS.X, T6112XA.X, T6112XD.X, T6112XS.X, T61772A.X, T61772D.X, T61772S.X, T61782A.X, T61782D.X, T61782S.X, T618X2A.X, T618X2D.X, T618X2S.X, T6192XA.X, T6192XD.X, T6192XS.X, T620X2A.X, T620X2D.X, T620X2S.X, T621X2A.X, T621X2D.X, T621X2S.X, T622X2A.X, T622X2D.X, T622X2S.X, T628X2A.X, T628X2D.X, T628X2S.X, T6292XA.X, T6292XD.X, T6292XS.X, T63002A.X, T63002D.X, T63002S.X, T63012A.X, T63012D.X, T63012S.X, T63022A.X, T63022D.X, T63022S.X, T63032A.X, T63032D.X, T63032S.X, T63042A.X, T63042D.X, T63042S.X, T63062A.X, T63062D.X, T63062S.X, T63072A.X, T63072D.X, T63072S.X, T63082A.X, T63082D.X, T63082S.X, T63092A.X, T63092D.X, T63092S.X, T63112A.X, T63112D.X, T63112S.X, T63122A.X, T63122D.X, T63122S.X, T63192A.X, T63192D.X, T63192S.X, T632X2A.X, T632X2D.X, T632X2S.X, T63302A.X, T63302D.X, T63302S.X, T63312A.X, T63312D.X, T63312S.X, T63322A.X, T63322D.X, T63322S.X, T63332A.X, T63332D.X, T63332S.X, T63392A.X, T63392D.X, T63392S.X, T63412A.X, T63412D.X, T63412S.X, T63422A.X, T63422D.X, T63422S.X, T63432A.X, T63432D.X, T63432S.X, T63442A.X, T63442D.X, T63442S.X, T63452A.X, T63452D.X, T63452S.X, T63462A.X, T63462D.X, T63462S.X, T63482A.X, T63482D.X, T63482S.X, T63512A.X, T63512D.X, T63512S.X, T63592A.X, T63592D.X, T63592S.X, T63612A.X, T63612D.X, T63612S.X, T63622A.X, T63622D.X, T63622S.X, T63632A.X, T63632D.X, T63632S.X, T63692A.X, T63692D.X, T63692S.X, T63712A.X, T63712D.X, T63712S.X, T63792A.X, T63792D.X, T63792S.X, T63812A.X, T63812D.X, T63812S.X, T63822A.X, T63822D.X, T63822S.X, T63832A.X, T63832D.X, T63832S.X, T63892A.X, T63892D.X, T63892S.X, T6392XA.X, T6392XD.X, T6392XS.X, T6402XA.X, T6402XD.X, T6402XS.X, T6482XA.X, T6482XD.X, T6482XS.X, T650X2A.X, T650X2D.X, T650X2S.X, T651X2A.X, T651X2D.X, T651X2S.X, T65212A.X, T65212D.X, T65212S.X, T65222A.X, T65222D.X, T65222S.X, T65292A.X, T65292D.X, T65292S.X, T653X2A.X, T653X2D.X, T653X2S.X, T654X2A.X, T654X2D.X, T654X2S.X,

		T655X2A.X, T655X2D.X, T655X2S.X, T656X2A.X, T656X2D.X, T656X2S.X, T65812A.X, T65812D.X, T65812S.X, T65822A.X, T65822D.X, T65822S.X, T65832A.X, T65832D.X, T65832S.X, T65892A.X, T65892D.X, T65892S.X, T6592XA.X, T6592XD.X, T6592XS.X, T71112A.X, T71112D.X, T71112S.X, T71122A.X, T71122D.X, T71122S.X, T71132A.X, T71132D.X, T71132S.X, T71152A.X, T71152D.X, T71152S.X, T71162A.X, T71162D.X, T71162S.X, T71192A.X, T71192D.X, T71192S.X, T71222A.X, T71222D.X, T71222S.X, T71232A.X, T71232D.X, T71232S.X, X710XXA.X, X710XXD.X, X710XXS.X, X711XXA.X, X711XXD.X, X711XXS.X, X712XXA.X, X712XXD.X, X712XXS.X, X713XXA.X, X713XXD.X, X713XXS.X, X718XXA.X, X718XXD.X, X718XXS.X, X719XXA.X, X719XXD.X, X719XXS.X, X72XXXA.X, X72XXXD.X, X72XXXS.X, X730XXA.X, X730XXD.X, X730XXS.X, X731XXA.X, X731XXD.X, X731XXS.X, X732XXA.X, X732XXD.X, X732XXS.X, X738XXA.X, X738XXD.X, X738XXS.X, X739XXA.X, X739XXD.X, X739XXS.X, X7401XA.X, X7401XD.X, X7401XS.X, X7402XA.X, X7402XD.X, X7402XS.X, X7409XA.X, X7409XD.X, X7409XS.X, X748XXA.X, X748XXD.X, X748XXS.X, X749XXA.X, X749XXD.X, X749XXS.X, X75XXXA.X, X75XXXD.X, X75XXXS.X, X76XXXA.X, X76XXXD.X, X76XXXS.X, X770XXA.X, X770XXD.X, X770XXS.X, X771XXA.X, X771XXD.X, X771XXS.X, X772XXA.X, X772XXD.X, X772XXS.X, X773XXA.X, X773XXD.X, X773XXS.X, X778XXA.X, X778XXD.X, X778XXS.X, X779XXA.X, X779XXD.X, X779XXS.X, X780XXA.X, X780XXD.X, X780XXS.X, X781XXA.X, X781XXD.X, X781XXS.X, X782XXA.X, X782XXD.X, X782XXS.X, X788XXA.X, X788XXD.X, X788XXS.X, X789XXA.X, X789XXD.X, X789XXS.X, X79XXXA.X, X79XXXD.X, X79XXXS.X, X80XXXA.X, X80XXXD.X, X80XXXS.X, X810XXA.X, X810XXD.X, X810XXS.X, X811XXA.X, X811XXD.X, X811XXS.X, X818XXA.X, X818XXD.X, X818XXS.X, X820XXA.X, X820XXD.X, X820XXS.X, X821XXA.X, X821XXD.X, X821XXS.X, X822XXA.X, X822XXD.X, X822XXS.X, X828XXA.X, X828XXD.X, X828XXS.X, X830XXA.X, X830XXD.X, X830XXS.X, X831XXA.X, X831XXD.X, X831XXS.X, X832XXA.X, X832XXD.X, X832XXS.X, X838XXA.X, X838XXD.X, X838XXS.X, Z915.X
Misc disorders	293.9, 300.11, 300.12, 300.13, 300.14, 300.15, 300.16, 300.19, 300.6, 300.7, 300.81, 300.82, 302.1, 302.2, 302.3, 302.4, 302.50, 302.51, 302.52, 302.53, 302.6, 302.70, 302.71, 302.72, 302.73, 302.74, 302.75, 302.76, 302.79, 302.81, 302.82, 302.83, 302.84, 302.85, 302.89, 302.9, 306.0, 306.1, 306.2, 306.3, 306.4, 306.50, 306.51, 306.52, 306.53, 306.59, 306.6, 306.7,	F061.X, F068.X, F440.X, F441.X, F442.X, F444.X, F445.X, F446.X, F447.X, F4481.X, F4489.X, F449.X, F450.X, F451.X, F4520.X, F4521.X, F4522.X, F4529.X, F4541.X, F4542.X, F458.X, F459.X, F481.X, F5000.X, F5001.X, F5002.X, F502.X, F508.X, F5081.X, F5082.X, F5089.X, F509.X, F5101.X, F5102.X, F5103.X, F5104.X, F5105.X, F5109.X, F5111.X, F5112.X, F5113.X, F5119.X, F513.X, F514.X, F515.X, F518.X, F519.X, F520.X, F521.X, F5221.X, F5222.X, F5231.X, F5232.X, F524.X, F525.X, F526.X, F528.X, F529.X, F53.X, F530.X, F531.X, F54.X, F59.X, F640.X, F641.X, F648.X, F649.X, F650.X, F651.X, F652.X, F653.X, F654.X, F6550.X, F6551.X,

	306.8, 306.9, 307.1, 307.40, 307.41, 307.42, 307.43, 307.44, 307.45, 307.46, 307.47, 307.48, 307.49, 307.50, 307.51, 307.52, 307.53, 307.54, 307.59, 307.80, 307.81, 307.89, 310.1, 316, 648.40, 648.41, 648.42, 648.43, 648.44, V40.2, V40.3, V40.31, V40.39, V40.9, V67.3	F6552.X, F6581.X, F6589.X, F659.X, F66.X, F6810.X, F6811.X, F6812.X, F6813.X, F688.X, F68A.X, F99.X, O906.X, R37.X, R4589.X, Z87890.X, Z9183.X
Back and neck pain	722.30, 722.32, 722.33, 722.70, 722.72, 722.73, 722.80, 722.82, 722.83, 722.90, 722.92, 722.93, 737.1, 737.3, 738.4, 738.5, 739.2, 739.3, 739.4, 756.10, 756.11, 756.12, 756.19, 805.4, 805.8, 839.2, 839.42, 846, 846.0, 847.1, 847.2, 847.3, 847.9, 721.3x - 721.9x, 722.2x, 724.xx, 756.13, 721.0X, 721.1X, 722.0X, 722.31, 722.71, 722.81, 722.91, 723.XX, 839.0, 839.1, 847.0	M43.20, M43.21, M43.22, M43.23, M43.24, M43.25, M43.26, M43.27, M43.28, M43.6, M46.00, M46.01, M46.02, M46.03, M46.04, M46.05, M46.06, M46.07, M46.08, M46.09, M46.1, M46.30, M46.31, M46.32, M46.33, M46.34, M46.35, M46.36, M46.37, M46.38, M46.39, M46.40, M46.41, M46.42, M46.43, M46.44, M46.45, M46.46, M46.47, M46.48, M46.49, M46.50, M46.51, M46.52, M46.53, M46.54, M46.55, M46.56, M46.57, M46.58, M46.59, M46.80, M46.81, M46.82, M46.83, M46.84, M46.85, M46.86, M46.87, M46.88, M46.89, M46.90, M46.91, M46.92, M46.93, M46.94, M46.95, M46.96, M46.97, M46.98, M46.99, M47.011, M47.012, M47.013, M47.014, M47.015, M47.016, M47.019, M47.021, M47.022, M47.029, M47.10, M47.11, M47.12, M47.13, M47.14, M47.15, M47.16, M47.17, M47.18, M47.20, M47.21, M47.22, M47.23, M47.24, M47.25, M47.26, M47.27, M47.28, M47.811, M47.812, M47.813, M47.814, M47.815, M47.816, M47.817, M47.818, M47.819, M47.891, M47.892, M47.893, M47.894, M47.895, M47.896, M47.897, M47.898, M47.899, M47.9, M48.00, M48.01, M48.02, M48.03, M48.04, M48.05, M48.06, M48.061, M48.062, M48.07, M48.08, M48.10, M48.11, M48.12, M48.13, M48.14, M48.15, M48.16, M48.17, M48.18, M48.19, M48.20, M48.21, M48.22, M48.23, M48.24, M48.25, M48.26, M48.27, M48.30, M48.31, M48.32, M48.33, M48.34, M48.35, M48.36, M48.37, M48.38, M48.9, M49.80, M49.81, M49.82, M49.83, M49.84, M49.85, M49.86, M49.87, M49.88, M49.89, M50.00, M50.01, M50.02, M50.020, M50.021, M50.022, M50.023, M50.03, M50.10, M50.11, M50.12, M50.120, M50.121, M50.122, M50.123, M50.13, M50.20, M50.21, M50.22, M50.220, M50.221, M50.222, M50.223, M50.23, M50.30, M50.31, M50.32, M50.320, M50.321, M50.322, M50.323, M50.33, M50.80, M50.81, M50.82, M50.820, M50.821, M50.822, M50.823, M50.83, M50.90, M50.91, M50.92, M50.920, M50.921, M50.922, M50.923, M50.93, M51.04, M51.05, M51.06, M51.07, M51.14, M51.15, M51.16, M51.17, M51.24, M51.25, M51.26, M51.27, M51.34, M51.35, M51.36, M51.37, M51.44, M51.45, M51.46, M51.47, M51.84, M51.85, M51.86, M51.87, M51.9, M53.0, M53.1, M53.2X1, M53.2X2, M53.2X3, M53.2X4, M53.2X5, M53.2X6, M53.2X7, M53.2X8, M53.2X9, M53.3, M53.80, M53.81, M53.82, M53.83, M53.84, M53.85, M53.86, M53.87, M53.88, M53.9, M54.00, M54.01, M54.02, M54.03, M54.04, M54.05, M54.06, M54.07, M54.08, M54.09, M54.10, M54.11, M54.12, M54.13, M54.14,

		M54.15, M54.16, M54.17, M54.18, M54.2, M54.30, M54.31, M54.32, M54.40, M54.41, M54.42, M54.5, M54.6, M54.81, M54.89, M54.9, M62.830, M96.1, M99.20, M99.21, M99.22, M99.23, M99.24, M99.25, M99.26, M99.27, M99.28, M99.29, M99.30, M99.31, M99.32, M99.33, M99.34, M99.35, M99.36, M99.37, M99.38, M99.39, M99.40, M99.41, M99.42, M99.43, M99.44, M99.45, M99.46, M99.47, M99.48, M99.49, M99.50, M99.51, M99.52, M99.53, M99.54, M99.55, M99.56, M99.57, M99.58, M99.59, M99.60, M99.61, M99.62, M99.63, M99.64, M99.65, M99.66, M99.67, M99.68, M99.69, M99.70, M99.71, M99.72, M99.73, M99.74, M99.75, M99.76, M99.77, M99.78, M99.79, P11.5, S33.30XA, S33.30XD, S33.30XS, S33.39XA, S33.39XD, S33.39XS, M43.09, M43.19, M42.00, M42.01, M42.02, M42.03, M42.04, M42.05, M42.06, M42.07, M42.08, M42.09, M42.10, M42.11, M42.12, M42.13, M42.14, M42.15, M42.16, M42.17, M42.18, M42.19, Q67.5, Q76.49, Q77.8, Q77.9, S32.9XXA, S32.9XXB, S32.9XXS, S14.2XXA, S14.2XXD, S14.2XXS, S24.2XXA, S24.2XXD, S24.2XXS, S34.21XA, S34.21XD, S34.21XS, S34.22XA, S34.22XD, S34.22XS, M45.0, M45.9, S13.4XXA, S13.4XXD, S13.4XXS, S23.3XXA, S23.3XXD, S23.3XXS, S33.5XXA, S33.5XXD, S33.5XXS, S33.8XXA, S33.8XXD, S33.8XXS, S33.9XXA, S33.9XXD, S33.9XXS, S13.0XXA, S13.0XXD, S13.0XXS, S23.0XXA, S23.0XXD, S23.0XXS, S33.0XXA, S33.0XXD, S33.0XXS, S17.8XXA, S17.8XXD, S17.8XXS, S17.9XXA, S17.9XXD, S17.9XXS, S13.20XA, S13.20XD, S13.20XS, S13.29XA, S13.29XD, S13.29XS, M95.3, Q18.3, Q18.8, Q18.9, Z87.790, S12.8XXA, S12.8XXD, S12.8XXS, S12.9XXA, S12.9XXD, S12.9XXS, S14.4XXA, S14.4XXD, S14.4XXS, S14.8XXA, S14.8XXD, S14.8XXS, S14.9XXA, S14.9XXD, S14.9XXS, S16.8XXA, S16.8XXD, S16.8XXS, S16.9XXA, S16.9XXD, S16.9XXS, S19.80XA, S19.80XD, S19.80XS, S19.89XA, S19.89XD, S19.89XS, S19.9XXA, S19.9XXD, S19.9XXS, M54.00, M54.01, M54.02, M54.03, M54.04, M54.05, M54.06, M54.07, M54.08, M54.09, S13.8XXA, S13.8XXD, S13.8XXS, S13.9XXA, S13.9XXD, S13.9XXS, S16.1XXA, S16.1XXD, S16.1XXS

Joint disorders	711.XX, 712.XX, 713.X, 714.XX, 715.XX, 716.XX, 717.XX, 718.XX, 719.XX, 725, 726.XX, 727.XX, 728.XX, 729.3X, 729.7X, 729.8X, 729.9X, 730.XX, 731.X, 732.X, 733.XX, 734, 735.X, 736.XX, 737.2X, 737.4X, 738.1X, 710, 710.1, 710.3, 710.4, 710.5, 710.8, 710.9, 729, 729.2, 729.4, 729.5, 729.6, 737, 737.8, 737.9, 738, 738.2, 738.3, 738.6, 738.7, 738.8, 738.9, 739, 739.1, 739.5, 739.6, 739.7, 739.8, 739.9	M15.0, M15.1, M15.2, M15.3, M15.4, M15.8, M15.9, M16.0, M16.10, M16.11, M16.12, M16.2, M16.30, M16.31, M16.32, M16.4, M16.50, M16.51, M16.52, M16.6, M16.7, M16.9, M17.0, M17.10, M17.11, M17.12, M17.2, M17.30, M17.31, M17.32, M17.4, M17.5, M17.9, M18.0, M18.10, M18.11, M18.12, M18.2, M18.30, M18.31, M18.32, M18.4, M18.50, M18.51, M18.52, M18.9, M19.011, M19.012, M19.019, M19.021, M19.022, M19.029, M19.031, M19.032, M19.039, M19.041, M19.042, M19.049, M19.071, M19.072, M19.079, M19.111, M19.112, M19.119, M19.121, M19.122, M19.129, M19.131, M19.132, M19.139, M19.141, M19.142, M19.149, M19.171, M19.172, M19.179, M19.211, M19.212, M19.219, M19.221, M19.222, M19.229, M19.231, M19.232, M19.239, M19.241, M19.242, M19.249, M19.271, M19.272, M19.279, M19.90, M19.91, M19.92, M19.93, M02.00, M02.011, M02.012, M02.019, M02.021, M02.022, M02.029, M02.031, M02.032, M02.039, M02.041, M02.042, M02.049, M02.051, M02.052, M02.059, M02.061, M02.062, M02.069, M02.071, M02.072, M02.079, M02.08, M02.09, M02.20, M02.211, M02.212, M02.219, M02.221, M02.222, M02.229, M02.231, M02.232, M02.239, M02.241, M02.242, M02.249, M02.251, M02.252, M02.259, M02.261, M02.262, M02.269, M02.271, M02.272, M02.279, M02.28, M02.29, M07.60, M07.611, M07.612, M07.619, M07.621, M07.622, M07.629, M07.631, M07.632, M07.639, M07.641, M07.642, M07.649, M07.651, M07.652, M07.659, M07.661, M07.662, M07.669, M07.671, M07.672, M07.679, M07.68, M07.69, M12.10, M12.111, M12.112, M12.119, M12.121, M12.122, M12.129, M12.131, M12.132, M12.139, M12.141, M12.142, M12.149, M12.151, M12.152, M12.159, M12.161, M12.162, M12.169, M12.171, M12.172, M12.179, M12.18, M12.19, M12.20, M12.211, M12.212, M12.219, M12.221, M12.222, M12.229, M12.231, M12.232, M12.239, M12.241, M12.242, M12.249, M12.251, M12.252, M12.259, M12.261, M12.262, M12.269, M12.271, M12.272, M12.279, M12.28, M12.29, M12.30, M12.311, M12.312, M12.319, M12.321, M12.322, M12.329, M12.331, M12.332, M12.339, M12.341, M12.342, M12.349, M12.351, M12.352, M12.359, M12.361, M12.362, M12.369, M12.371, M12.372, M12.379, M12.38, M12.39, M12.40, M12.411, M12.412, M12.419, M12.421, M12.422, M12.429, M12.431, M12.432, M12.439, M12.441, M12.442, M12.449, M12.451, M12.452, M12.459, M12.461, M12.462, M12.469, M12.471, M12.472, M12.479, M12.48, M12.49, M12.80, M12.811, M12.812, M12.819, M12.821, M12.822, M12.829, M12.831, M12.832, M12.839, M12.841, M12.842, M12.849, M12.851, M12.852, M12.859, M12.861, M12.862, M12.869, M12.871, M12.872, M12.879, M12.88, M12.89, M12.9, M13.0, M13.10, M13.111, M13.112, M13.119, M13.121, M13.122, M13.129, M13.131, M13.132, M13.139, M13.141, M13.142, M13.149, M13.151, M13.152, M13.159,

		M13.161, M13.162, M13.169, M13.171, M13.172, M13.179, M13.80, M13.811, M13.812, M13.819, M13.821, M13.822, M13.829, M13.831, M13.832, M13.839, M13.841, M13.842, M13.849, M13.851, M13.852, M13.859, M13.861, M13.862, M13.869, M13.871, M13.872, M13.879, M13.88, M13.89, M14.60, M14.611, M14.612, M14.619, M14.621, M14.622, M14.629, M14.631, M14.632, M14.639, M14.641, M14.642, M14.649, M14.651, M14.652, M14.659, M14.661, M14.662, M14.669, M14.671, M14.672, M14.679, M14.68, M14.69, M14.80, M14.811, M14.812, M14.819, M14.821, M14.822, M14.829, M14.831, M14.832, M14.839, M14.841, M14.842, M14.849, M14.851, M14.852, M14.859, M14.861, M14.862, M14.869, M14.871, M14.872, M14.879, M14.88, M14.89, M24.00, M24.011, M24.012, M24.019, M24.021, M24.022, M24.029, M24.031, M24.032, M24.039, M24.041, M24.042, M24.049, M24.051, M24.052, M24.059, M24.071, M24.072, M24.073, M24.074, M24.075, M24.076, M24.08, M24.30, M24.311, M24.312, M24.319, M24.321, M24.322, M24.329, M24.331, M24.332, M24.339, M24.341, M24.342, M24.349, M24.351, M24.352, M24.359, M24.361, M24.362, M24.369, M24.371, M24.372, M24.373, M24.374, M24.375, M24.376, M24.60, M24.611, M24.612, M24.619, M24.621, M24.622, M24.629, M24.631, M24.632, M24.639, M24.641, M24.642, M24.649, M24.651, M24.652, M24.659, M24.661, M24.662, M24.669, M24.671, M24.672, M24.673, M24.674, M24.675, M24.676, M24.7, M24.80, M24.811, M24.812, M24.819, M24.821, M24.822, M24.829, M24.831, M24.832, M24.839, M24.841, M24.842, M24.849, M24.851, M24.852, M24.859, M24.871, M24.872, M24.873, M24.874, M24.875, M24.876, M24.9, M25.00, M25.011, M25.012, M25.019, M25.021, M25.022, M25.029, M25.031, M25.032, M25.039, M25.041, M25.042, M25.049, M25.051, M25.052, M25.059, M25.061, M25.062, M25.069, M25.071, M25.072, M25.073, M25.074, M25.075, M25.076, M25.08, M25.10, M25.111, M25.112, M25.119, M25.121, M25.122, M25.129, M25.131, M25.132, M25.139, M25.141, M25.142, M25.149, M25.151, M25.152, M25.159, M25.161, M25.162, M25.169, M25.171, M25.172, M25.173, M25.174, M25.175, M25.176, M25.18, M25.20, M25.211, M25.212, M25.219, M25.221, M25.222, M25.229, M25.231, M25.232, M25.239, M25.241, M25.242, M25.249, M25.251, M25.252, M25.259, M25.261, M25.262, M25.269, M25.271, M25.272, M25.279, M25.28, M25.30, M25.311, M25.312, M25.319, M25.321, M25.322, M25.329, M25.331, M25.332, M25.339, M25.341, M25.342, M25.349, M25.351, M25.352, M25.359, M25.361, M25.362, M25.369, M25.371, M25.372, M25.373, M25.374, M25.375, M25.376, M25.40, M25.411, M25.412, M25.419, M25.421, M25.422, M25.429, M25.431, M25.432, M25.439, M25.441, M25.442, M25.449, M25.451, M25.452, M25.459, M25.461, M25.462, M25.469, M25.471,

		M25.472, M25.473, M25.474, M25.475, M25.476, M25.48, M25.50, M25.511, M25.512, M25.519, M25.521, M25.522, M25.529, M25.531, M25.532, M25.539, M25.541, M25.542, M25.549, M25.551, M25.552, M25.559, M25.561, M25.562, M25.569, M25.571, M25.572, M25.579, M25.60, M25.611, M25.612, M25.619, M25.621, M25.622, M25.629, M25.631, M25.632, M25.639, M25.641, M25.642, M25.649, M25.651, M25.652, M25.659, M25.661, M25.662, M25.669, M25.671, M25.672, M25.673, M25.674, M25.675, M25.676, M25.70, M25.711, M25.712, M25.719, M25.721, M25.722, M25.729, M25.731, M25.732, M25.739, M25.741, M25.742, M25.749, M25.751, M25.752, M25.759, M25.761, M25.762, M25.769, M25.771, M25.772, M25.773, M25.774, M25.775, M25.776, M25.78, M25.80, M25.811, M25.812, M25.819, M25.821, M25.822, M25.829, M25.831, M25.832, M25.839, M25.841, M25.842, M25.849, M25.851, M25.852, M25.859, M25.861, M25.862, M25.869, M25.871, M25.872, M25.879, M25.9, M36.1, M36.2, M36.3, M36.4, R29.4, M05.00, M05.011, M05.012, M05.019, M05.021, M05.022, M05.029, M05.031, M05.032, M05.039, M05.041, M05.042, M05.049, M05.051, M05.052, M05.059, M05.061, M05.062, M05.069, M05.071, M05.072, M05.079, M05.09, M05.10, M05.111, M05.112, M05.119, M05.121, M05.122, M05.129, M05.131, M05.132, M05.139, M05.141, M05.142, M05.149, M05.151, M05.152, M05.159, M05.161, M05.162, M05.169, M05.171, M05.172, M05.179, M05.19, M05.20, M05.211, M05.212, M05.219, M05.221, M05.222, M05.229, M05.231, M05.232, M05.239, M05.241, M05.242, M05.249, M05.251, M05.252, M05.259, M05.261, M05.262, M05.269, M05.271, M05.272, M05.279, M05.29, M05.30, M05.311, M05.312, M05.319, M05.321, M05.322, M05.329, M05.331, M05.332, M05.339, M05.341, M05.342, M05.349, M05.351, M05.352, M05.359, M05.361, M05.362, M05.369, M05.371, M05.372, M05.379, M05.39, M05.40, M05.411, M05.412, M05.419, M05.421, M05.422, M05.429, M05.431, M05.432, M05.439, M05.441, M05.442, M05.449, M05.451, M05.452, M05.459, M05.461, M05.462, M05.469, M05.471, M05.472, M05.479, M05.49, M05.50, M05.511, M05.512, M05.519, M05.521, M05.522, M05.529, M05.531, M05.532, M05.539, M05.541, M05.542, M05.549, M05.551, M05.552, M05.559, M05.561, M05.562, M05.569, M05.571, M05.572, M05.579, M05.59, M05.60, M05.611, M05.612, M05.619, M05.621, M05.622, M05.629, M05.631, M05.632, M05.639, M05.641, M05.642, M05.649, M05.651, M05.652, M05.659, M05.661, M05.662, M05.669, M05.671, M05.672, M05.679, M05.69, M05.70, M05.711, M05.712, M05.719, M05.721, M05.722, M05.729, M05.731, M05.732, M05.739, M05.741, M05.742, M05.749, M05.751, M05.752, M05.759, M05.761, M05.762, M05.769, M05.771, M05.772, M05.779, M05.79, M05.80, M05.811, M05.812, M05.819, M05.821, M05.822, M05.829,

		M05.831, M05.832, M05.839, M05.841, M05.842, M05.849, M05.851, M05.852, M05.859, M05.861, M05.862, M05.869, M05.871, M05.872, M05.879, M05.89, M05.9, M06.00, M06.011, M06.012, M06.019, M06.021, M06.022, M06.029, M06.031, M06.032, M06.039, M06.041, M06.042, M06.049, M06.051, M06.052, M06.059, M06.061, M06.062, M06.069, M06.071, M06.072, M06.079, M06.08, M06.09, M06.1, M06.20, M06.211, M06.212, M06.219, M06.221, M06.222, M06.229, M06.231, M06.232, M06.239, M06.241, M06.242, M06.249, M06.251, M06.252, M06.259, M06.261, M06.262, M06.269, M06.271, M06.272, M06.279, M06.28, M06.29, M06.30, M06.311, M06.312, M06.319, M06.321, M06.322, M06.329, M06.331, M06.332, M06.339, M06.341, M06.342, M06.349, M06.351, M06.352, M06.359, M06.361, M06.362, M06.369, M06.371, M06.372, M06.379, M06.38, M06.39, M06.4, M06.80, M06.811, M06.812, M06.819, M06.821, M06.822, M06.829, M06.831, M06.832, M06.839, M06.841, M06.842, M06.849, M06.851, M06.852, M06.859, M06.861, M06.862, M06.869, M06.871, M06.872, M06.879, M06.88, M06.89, M06.9, M08.00, M08.011, M08.012, M08.019, M08.021, M08.022, M08.029, M08.031, M08.032, M08.039, M08.041, M08.042, M08.049, M08.051, M08.052, M08.059, M08.061, M08.062, M08.069, M08.071, M08.072, M08.079, M08.08, M08.09, M08.1, M08.20, M08.211, M08.212, M08.219, M08.221, M08.222, M08.229, M08.231, M08.232, M08.239, M08.241, M08.242, M08.249, M08.251, M08.252, M08.259, M08.261, M08.262, M08.269, M08.271, M08.272, M08.279, M08.28, M08.29, M08.3, M08.40, M08.411, M08.412, M08.419, M08.421, M08.422, M08.429, M08.431, M08.432, M08.439, M08.441, M08.442, M08.449, M08.451, M08.452, M08.459, M08.461, M08.462, M08.469, M08.471, M08.472, M08.479, M08.48, M08.80, M08.811, M08.812, M08.819, M08.821, M08.822, M08.829, M08.831, M08.832, M08.839, M08.841, M08.842, M08.849, M08.851, M08.852, M08.859, M08.861, M08.862, M08.869, M08.871, M08.872, M08.879, M08.88, M08.89, M08.90, M08.911, M08.912, M08.919, M08.921, M08.922, M08.929, M08.931, M08.932, M08.939, M08.941, M08.942, M08.949, M08.951, M08.952, M08.959, M08.961, M08.962, M08.969, M08.971, M08.972, M08.979, M08.98, M08.99, M12.00, M12.011, M12.012, M12.019, M12.021, M12.022, M12.029, M12.031, M12.032, M12.039, M12.041, M12.042, M12.049, M12.051, M12.052, M12.059, M12.061, M12.062, M12.069, M12.071, M12.072, M12.079, M12.08, M12.09, M45.0, M45.1, M45.2, M45.3, M45.4, M45.5, M45.6, M45.7, M45.8, M45.9, M48.8X1, M48.8X2, M48.8X3, M48.8X4, M48.8X5, M48.8X6, M48.8X7, M48.8X8, M48.8X9

Supplemental Table 2. Estimated Age-adjusted Death Rate (per 100,000 persons) due to drug poisoning, 13 Categories (in ranges) (n=88 counties)

CDCDRUGPOISON_DEATH_RATE	Frequency	Percent
**6.1-8**	1	1.14
**8.1-10**	7	7.95
**10.1-12**	9	10.23
**12.1-14**	14	15.91
**14.1-16**	12	13.64
**16.1-18**	10	11.36
**18.1-20**	8	9.09
**20.1-22**	7	7.95
**22.1-24**	5	5.68
**24.1-26**	5	5.68
**26.1-28**	3	3.41
**28.1-30**	4	4.55
**>30**	3	3.41

Reference: Centers for Disease Control and Prevention:
https://www.cdc.gov/drugoverdose/data/statedeaths.html

Supplemental Table 3. The distribution of surgery procedure groups and the incidence of new persistent opioid use by group.

Surgery Procedure Group	Sample Size	Persistent Opioid Use, N (%)
auditory system	22	1 (4.6%)
cardiovascular system	231	15 (6.5%)
digestive system	1196	80 (6.7%)
endocrine system	64	2 (3.1%)
eye and ocular adnexa	45	6 (13.3%)
female genital system	319	11 (3.4%)
general	6	0 (0%)
hemic and lymphatic systems	12	0 (0%)
integumentary system	514	31 (6.0%)
male genital system	103	7 (6.8%)
maternity care and delivery	111	6 (5.4%)
multiple surgery groups	499	22 (4.4%)
musculoskeletal system	631	42 (6.7%)
nervous system	158	9 (5.7%)
other	2	0 (0%)
respiratory system	89	8 (9.0%)
urinary system	114	5 (4.4%)
**Total**	4116	245 (6.0%)

Supplemental Table 4. Logistic regression coefficients in the prediction model equations for surgical patients becoming a persistent opioid user 90 to 180 days after surgery based on logistic regression analysis using 20 multiple imputations based on both EHR and claims data (n=4116)

Covariate, parameter[a]	Model E+C		Model E+C+G	
	estimate	std. error	estimate	std. error
Intercept, β_0	-5.756972	0.853573	-9.817036	1.249766
Male, β_1	0.010626	0.140756	0.013187	0.141724
Black[b], β_2	0.287836	0.207869	0.269913	0.211695
Other race[b], β_3	0.019349	0.280801	0.057410	0.282912
Age[4], β_4	0.070607	0.038092	0.077687	0.038247
Age-squared, β_5	-0.000637	0.000421	-0.000720	0.000423
Ever smoker, β_6	0.262275	0.138428	0.230244	0.139537
Pain Score[4], β_7	0.101479	0.105641	0.109724	0.106801
Pain Score squared, β_8	-0.001799	0.012716	-0.003068	0.012847
Benzodiazepine Use, β_9	0.164958	0.187896	0.168510	0.188264
Back and neck pain, β_{10}	0.619033	0.138265	0.600882	0.139011
Joint disorders, β_{11}	0.451648	0.148437	0.456340	0.149105
Mood disorders, β_{12}	0.525897	0.147398	0.535962	0.148050
Unemployment rate, β_{13}			0.098823	0.066141
Unemployment rate squared, β_{14}			-0.005789	0.003702
Opioid retail prescriptions rate[c], β_{15}			0.036554	0.016526
Opioid retail prescriptions rate squared, β_{16}			-0.000191	0.000095
Drug poison death rated[d], β_{17}			0.283950	0.101120
Drug poison death rate squared, β_{18}			-0.008482	0.003073

[a]The prediction model equations based on the estimates of $(\beta_0, \ldots, \beta_{18})$ shown in this table are presented in the "Supplemental Methods" text. [b]reference is white race; [c]rate per 100 persons; [d]estimated age-adjusted death rate per 100 persons.

Supplemental Table 5. Patient characteristics and comorbid conditions based on EHR only and incidence of persistent opioid use 90 to 180 post-surgery (n=4116)

	Total number (column percent)	Persistent opioid use, No. (% for row subgroup)	p-value[2]
Benzodiazepine Use			0.55
No	3929 (95.5%)	232 (5.9%)	
Yes	187 (4.5%)	13 (7.0%)	
Back and neck pain			0.018
No	3680 (89.4%)	208 (5.7%)	
Yes	436 (10.6%)	37 (8.5%)	
Joint disorders			0.006
No	3228 (78.4%)	175 (5.4%)	
Yes	888 (21.6%)	70 (7.9%)	
Mood disorders			0.0011
No	3601 (87.5%)	198 (5.5%)	
Yes	515 (12.5%)	47 (9.1%)	
Suicidality			0.35*
No	4109 (99.8%)	244 (5.9%)	
Yes	7 (0.2%)	1 (14.3%)	
Disruptive behavior disorders			0.63*
No	4035 (98.0%)	242 (6.0%)	
Yes	81 (2.0%)	3 (3.7%)	
Personality disorders and schizophrenia			1.00*
No	4113 (99.9%)	245 (6.0%)	
Yes	3 (0.1%)	0 (0%)	
Substance use disorders			0.63
No	3812 (92.6%)	225 (5.9%)	
Yes	304 (7.4%)	20 (6.6%)	
Miscellaneous disorders			0.15*
No	4059 (98.6%)	239 (5.9%)	
Yes	57 (1.4%)	6 (10.5%)	

[1]based on EHR only; [2]Tests of whether patient characteristic or condition type is associated with new persistent opioid use: *Fisher's exact test; otherwise Pearson chi-square test.

Supplemental Table 6. Comparison of model results between datasets when patient characteristics and comorbid conditions are defined from electronic health records (EHR) only and EHR plus claims: Odds ratios (95% confidence intervals) for surgical patients becoming a persistent opioid user 90 to 180 days after surgery based on logistic regression analysis using 20 multiple imputations[1] (n=4116)

Covariate	EHR data only	EHR and Claims data[2]
Male	0.88 (0.67, 1.16)	1.01 (0.77, 1.33)
Black[3]	1.15 (0.77, 1.72)	1.33 (0.89, 2.00)
Other race[3]	0.90 (0.52, 1.55)	1.02 (0.59, 1.77)
Age[4]	1.09 (1.01, 1.17)*	1.07 (1.00, 1.16)
Age-squared	1.00 (1.00, 1.00)	1.00 (1.00, 1.00)
Ever smoker	1.37 (1.04, 1.79)*	1.30 (0.99, 1.71)
Pain Score[4]	1.11 (0.91, 1.36)	1.11 (0.90, 1.36)
Pain Score squared	1.00 (0.97, 1.02)	1.00 (0.97, 1.02)
Benzodiazepine Use	1.04 (0.57, 1.88)	1.18 (0.82, 1.70)
Back and neck pain	1.25 (0.86, 1.83)	1.86 (1.42, 2.44)***
Joint disorders	1.35 (1.00, 1.82)*	1.57 (1.17, 2.10)**
Mood disorders	1.49 (1.05, 2.11)*	1.69 (1.27, 2.26)***

[1]intercept estimate (standard error) is -5.45 (0.85) and -5.76 (0.85) for model fitted to EHR dataset (electronic health records) and dataset of EHR and claims, respectively. [2]These results are the same as for model E+C presented in Table 3 of the main article; [3]reference=white; [4]With inclusion of quadratic effects, these are approximate odds ratios for an unit increase in the continuous variable; *p < 0.05; ** p< 0.01; ***p<0.001

Supplemental Table 7. Average prediction performance of logistic regression model including patient characteristics and comorbid conditions for new persistent opioid users (n=4116) based on 1000 Monte-Carlo cross-validation samples. The 1st row cell entry for each model is the prediction performance statistic for predicting opioid use incidence calculated directly from the logistic model for incident opioid use based on 4116 participants. The 2nd row cell entry is the prediction performance statistic, which is the average of 1000 Monte-Carlo samples. The 3rd row cell entry is the Monte-Carlo cross-validation 95% CI for the prediction performance statistic.

Data Source[1]	AUC	Brier score	Classification table with cutoff point[2] of 0.06 for probability of opioid use			
			Sensitivity	Specificity	False positives	False negatives
EHR only	0.627	0.055	53.8	60.7	92.0	4.6
	0.593	0.056	51.8	60.9	92.3	4.8
	(0.538, 0.643)	(0.047, 0.065)	(41.1, 61.5)	(51.7, 68.9)	(91.3, 93.3)	(4.1, 5.5)
EHR+Claims[3]	0.676	0.054	58.7	65.0	90.4	3.9
	0.653	0.055	57.8	64.8	90.6	3.9
	(0.600, 0.706)	(0.047, 0.064)	(50.6, 64.2)	(59.1, 69.9)	(89.5, 91.7)	(3.4, 4.5)

[1]For both datasets, the 1st row cell entry is the average statistic across the 20 imputations; the 2nd and 3rd row cell entries are computed using a single imputation step for ordinal pain score for each Monte-Carlo sample replicate. [2]The cutoff point of 0.06 is determined by 245/4116=0.06. [3]Results for the EHR+Claims data is the same as for Model E+C in Table 4 of the main article.
